# L-Shaped Association of Serum Chloride Level With All-Cause and Cause-Specific Mortality in American Adults: Population-Based Prospective Cohort Study

**DOI:** 10.2196/49291

**Published:** 2023-11-13

**Authors:** Xinran Hou, Wei Xu, Chengliang Zhang, Zongbin Song, Maoen Zhu, Qulian Guo, Jian Wang

**Affiliations:** 1 Department of Anesthesiology Xiangya Hospital Central South University Changsha China; 2 National Clinical Research Center for Geriatric Disorders Xiangya Hospital Central South University Changsha China; 3 Department of Anesthesiology Hunan Provincial Maternal and Child Health Care Hospital University of South China Changsha China; 4 Department of Cardiovascular Surgery Xiangya Hospital Central South University Changsha China

**Keywords:** serum chloride, all-cause mortality, cause-specific mortality, National Health and Nutrition Examination Survey, National Death Index

## Abstract

**Background:**

Chloride is the most abundant anion in the human extracellular fluid and plays a crucial role in maintaining homeostasis. Previous studies have demonstrated that hypochloremia can act as an independent risk factor for adverse outcomes in various clinical settings. However, the association of variances of serum chloride with long-term mortality risk in general populations has been rarely investigated.

**Objective:**

This study aims to assess the association of serum chloride with all-cause and cause-specific mortality in the general American adult population.

**Methods:**

Data were collected from 10 survey cycles (1999-2018) of the National Health and Nutrition Examination Survey. All-cause mortality, cardiovascular disease (CVD) mortality, cancer mortality, and respiratory disease mortality data were obtained by linkage to the National Death Index through December 31, 2019. After adjusting for demographic factors and relevant lifestyle, laboratory items, and comorbid factors, weighted Cox proportional risk models were constructed to estimate hazard ratios and 95% CIs for all-cause and cause-specific mortality.

**Results:**

A total of 51,060 adult participants were included, and during a median follow-up of 111 months, 7582 deaths were documented, 2388 of CVD, 1639 of cancer, and 567 of respiratory disease. The weighted Kaplan-Meier survival analyses showed consistent highest mortality risk in individuals with the lowest quartiles of serum chloride. The multivariate-adjusted hazard ratios from lowest to highest quartiles of serum chloride (≤101.2, 101.3-103.2, 103.2-105.0, and ≥105.1 mmol/L) were 1.00 (95% CI reference), 0.77 (95% CI 0.67-0.89), 0.72 (95% CI 0.63-0.82), and 0.77 (95% CI 0.65-0.90), respectively, for all-cause mortality (*P* for linear trend<.001); 1.00 (95% CI reference), 0.63 (95% CI 0.51-0.79), 0.56 (95% CI 0.43-0.73), and 0.67 (95% CI 0.50-0.89) for CVD mortality (*P* for linear trend=.004); 1.00 (95% CI reference), 0.67 (95% CI 0.54-0.84), 0.65 (95% CI 0.50-0.85), and 0.65 (95% CI 0.48-0.87) for cancer mortality (*P* for linear trend=.004); and 1.00 (95% CI reference), 0.68 (95% CI 0.41-1.13), 0.59 (95% CI 0.40-0.88), and 0.51 (95% CI 0.31-0.84) for respiratory disease mortality (*P* for linear trend=.004). The restricted cubic spline analyses revealed the nonlinear and L-shaped associations of serum chloride with all-cause and cause-specific mortality (all *P* for nonlinearity<.05), in which lower serum chloride was prominently associated with higher mortality risk. The associations of serum chloride with mortality risk were robust, and no significant additional interaction effect was detected for all-cause mortality and CVD mortality (*P* for interaction>.05).

**Conclusions:**

In American adults, decreased serum chloride concentrations were independently associated with increased all-cause mortality, CVD mortality, cancer mortality, and respiratory disease mortality. Our findings suggested that serum chloride may serve as a promising cost-effective health indicator in the general adult population. Further studies are warranted to explore the potential pathophysiological mechanisms underlying the association between serum chloride and mortality.

## Introduction

### Background

Chloride is the most abundant anion in human extracellular fluid, accounting for approximately one-third of plasma tonicity and two-thirds of all plasma negative charges [1], and is predominantly confined to the extracellular fluid compartment [2]. Serum chloride plays an important role in many physiological processes in the body, including the maintenance of osmotic pressure, electroneutrality of body fluids, muscular activity, and regulation of blood pressure [3,4]. Serum chloride levels are predominantly regulated by the gastrointestinal tract and kidney. Chloride is secreted in the gastric juice as hydrochloric acid and can be absorbed by the entire intestine during digestion. In the United States, the daily intake of chloride for adult men is 7.8 to 11.8 g, whereas that for adult women is 5.8 to 7.8 g [5]. The primary source of chloride in the body is table salt (sodium chloride) in the diet as well as salt-containing foods [6]. The kidney can filter 19,440 mmol of chloride every day, 99.1% of which is reabsorbed, and only 180 mmol of chloride is excreted into urine, maintaining serum chloride within a narrow range [7].

Many clinical situations are related to the dysregulation of serum chloride. Serum chloride can be reduced either by chloride depletion, such as vomiting, diarrhea, burns, diuretic use, renal diseases, adrenal insufficiency, and metabolic alkalosis, or by water retention, such as excessive water intake, congestive heart failure, liver cirrhosis, and nephrotic syndrome. In contrast, serum chloride can be elevated by the loss of hypotonic fluids (eg, fever), administration of hypertonic fluid, or metabolic acidosis [2]. Chloride is a crucial constituent in diagnostic tests across numerous clinical scenarios. However, unlike other serum electrolytes such as sodium, potassium, and calcium, the importance of serum chloride is often overlooked despite its extensive measurement in routine blood biochemical tests. In recent years, serum chloride has exhibited a robust independent prognostic value, which seems even better than serum sodium, in patients with heart failure [8-11], chronic kidney disease (CKD) [12], and pulmonary arterial hypertension [13] and in patients who are critically ill [14-16]. For instance, in patients diagnosed with congestive heart failure, both hypochloremia and hyperchloremia have been associated with higher rates of mortality and hospitalization [11,17]. Similarly, lower chloride levels have been associated with an increased risk of mortality and progression to end-stage renal disease in patients with CKD [12,14]. Lower chloride level at 6 months after pulmonary arterial hypertension could predict long-term mortality [13]. In addition, research conducted on patients who are critically ill has demonstrated that both low and high levels of chloride are associated with higher rates of mortality and dysfunction in organs [14-16]. However, most studies were conducted among specific patient populations (eg, patients who were hospitalized or participants with heart disease or kidney disease), and researchers mainly focused on patients with hypochloremia or hyperchloremia. Knowledge of the relationship between the generally distributed serum chloride and mortality from the community-based population remains limited and requires further clarification. Understanding the potential association between chloride levels and mortality in the general population is essential for identifying individuals at higher risk and providing preventive strategies.

### Objective

To address this knowledge gap, we used a nationally representative cohort from the National Health and Nutrition Examination Survey (NHANES) 1999-2018 data set to investigate whether serum chloride was associated with all-cause and cause-specific mortality in the general American adult population.

## Methods

### Study Design and Participants Selection

The NHANES has been a continuous nationally representative survey investigating the health and nutritional status of the noninstitutionalized population of the United States since 1999 [18]. In this cohort study, we analyzed data in 10 cycles of NHANES from 1999-2018. Participants were restricted to those aged ≥18 years, not pregnant at the physical examination, and with eligible serum chloride measurement and mortality follow-up information.

### Ethical Considerations

All participants in the study signed a written informed consent form, and the NHANES data collection was approved by the Research Ethics Review Board of the National Center for Health Statistics [19]. The NHANES has strict protocols and procedures in place to ensure confidentiality and protect against identification. As our study was a secondary data analysis, which lacked personal identifiers, and the data in NHANES are freely accessible to the public on the web [20], it did not require review by an institutional review board [19].

### Measurement of Serum Chloride Concentration

Blood samples were collected and processed in the mobile examination center by certified laboratory professionals and then stored in biorepositories. Serum chloride was determined as part of the routine biochemistry profile with Beckman Synchron LX20 or Beckman Coulter UniCel DxC800 (Beckman Coulter, Inc), both of which used indirect (or diluted) ion-selective electrode methodology for the determination of serum chloride concentrations. Details about the laboratory procedure have been described on the NHANES website [20].

### Assessment of Covariates

The sociodemographic characteristics, namely, sex, age, race, education level, marital status, and family income-to-poverty ratio (PIR), were collected using standardized questionnaires during the household interview, as previously described [21].

Lifestyle factors were evaluated using a combination of data from physical examinations and questionnaires. BMI was calculated as weight (kg) divided by the square of height (m). Smoking status was classified as never (smoked <100 cigarettes/lifetime), former (≥100 cigarettes/lifetime but do not currently smoke), and current smokers. Alcohol users were classified as never (<12 drinks in a lifetime), former (≥12 drinks in a lifetime but did not drink in the last year), mild (≤1 drink/d for women or ≤2 drinks/d for men), moderate (≤2 drinks/d for women or ≤3 drinks/d for men), and heavy drinkers (>2 drinks/d for women or >3 drinks/d for men), where a drink referred to an alcoholic drink equivalent of 12 oz of beer, 4 oz of wine, or 1 oz of liquor (such as whiskey or gin) [22]. Diet quality was assessed by the Healthy Eating Index-2015 (HEI-2015), reflecting adherence to the 2015 to 2020 Dietary Guidelines for Americans [23]. Physical activity was quantified in metabolic equivalent tasks (METs) multiplied by exercise time (minutes) per week (METs min/wk) [24].

For laboratory variables, serum sodium, potassium, and bicarbonate levels were measured similarly to serum chloride levels, as described in the aforementioned method section. The estimated glomerular filtration rate (eGFR) was calculated using the CKD Epidemiology Collaboration equation (2009) [25].

Comorbid conditions were evaluated by combined information from NHANES data sets, as previously described [21]. Hypertension was determined by systolic blood pressure≥140 mm Hg, diastolic blood pressure ≥90 mm Hg, having been diagnosed by a physician, or current use of antihypertensive drugs. Diabetes was determined by fasting glucose level ≥7.0 mmol/L, random blood glucose level ≥11.1 mmol/L, Hemoglobin A1c level ≥6.5%, 2-hour glucose level ≥11.1 mmol/L after oral glucose tolerance tests, having been diagnosed by a physician or current use of the antidiabetic drug. Chronic obstructive pulmonary disease (COPD) was determined by first second of expiratory volume/forced vital capacity <0.7 after the use of bronchodilators in spirometry, having been diagnosed by a physician, or current use of drugs for COPD. Coronary heart disease (CHD), stroke, and cancer were assessed based on whether the participants had been diagnosed with these conditions by a doctor.

### Ascertainment of All-Cause and Cause-Specific Mortality

Mortality status for people who took part in the NHANES study was ascertained through probabilistic matching with the National Death Index Public-Use Linked Mortality Files up to December 31, 2019 [26]. The primary outcome was all-cause and cause-specific mortality, classified according to the international classification of diseases-10 codes recorded as the leading cause of death, in which cardiovascular disease (CVD) mortality corresponded to I00-I09, I11, I13, I20-I51, and I60-I69; cancer mortality corresponded to C00-C97; and respiratory disease mortality corresponded to J09-J18 and J40-J47.

In this study, the survival time has been calculated as months from the date of examination to the date of death or the end of follow-up (December 31, 2019), whichever came first.

### Statistical Analysis

Sample weights, clustering, and stratification were used in the data analyses to account for the stratified, multistage probability design of NHANES, in which the combined sample weight was calculated as 2/5×4-year sample weight for examinations in mobile examination centers for the period from 1999 to 2002 and 1/5×2-year sample weight for examinations in mobile examination centers for other survey cycles according to the NHANES analytic guidelines [27,28].

Data are presented as the survey-weighted mean (SE) for continuous variables or frequency (survey-weighted percentage) for categorical variables. Differences in baseline information were detected using survey-weighted ANOVA or chi-square test.

Survey-weighted Kaplan-Meier survival curves were plotted, and overall log-rank tests and pairwise comparisons between group levels with Benjamini and Hochberg corrections [29] were used to compare the cumulative survival in different serum chloride quartiles. Survey-weighted Cox proportional hazards models were used to estimate the hazard ratios (HRs) and 95% CIs for the association of serum chloride quartiles with all-cause and cause-specific mortality, with the first quartile as the reference, and the linear trend was evaluated by assigning a median value to each category as a continuous variable. The crude and multivariable models with different covariate adjustments were constructed to estimate mortality risk according to the Strengthening the Reporting of Observational Studies in Epidemiology (STROBE) reporting guideline [30], in which sex, age, and race were adjusted in model 1; education, marital status, PIR, BMI, smoking, alcohol use, HEI-2015, and physical activity were additionally adjusted in model 2; serum sodium, serum potassium, serum bicarbonate, eGFR, and use of diuretics as well as comorbidity of hypertension, diabetes, CHD, stroke, COPD, and cancer were additionally adjusted in model 3. Moreover, serum chloride was calculated as a continuous variable to estimate the mortality risk with each millimole per liter increase of serum chloride in different multivariate models. The proportion of missing data in the covariates was summarized, and in the primary analyses, the listwise deletion for missing variables was adopted for multivariate models.

To evaluate the potential nonlinear association of mortality with serum chloride, the restricted cubic spline analysis was performed in multivariable-adjusted models, in which the number of knots was determined based on the Akaike information criterion.

The subgroup analyses were performed according to the dichotomic stratified variables, namely, sex; age at recruitment; race; education level; marital status; PIR; BMI; smoker; drinker; HEI-2015; physical activity (physically active [≥600 MET-min/wk] vs physically inactive [<600 MET-min/wk]); and comorbidity of hypertension, diabetes, stroke, COPD, cancer, and CKD, determined by eGFR ≤60 mL/min/1.73 m^2^ or albuminuria ≥30 mg/g based on the Kidney Diseases Improving Global Outcomes guideline [31], using the fully adjusted model except for the specific stratification variable. The likelihood ratio test also inspected interactions of serum chloride with the stratification variables.

Robustness of the results was examined using several sensitivity analyses. First, participants who died within the first 2 years of follow-up were excluded to reduce potential reverse causation. Second, participants with extremely low or high serum chlorides (<1.5×IQR below the lower quartile or >1.5×IQR above the upper quartile) were excluded to test the effect of potential outliers. Third, participants with hypoalbuminemia (serum albumin level <35 g/L) were excluded to evaluate the impact of malnutrition on the relation between serum chloride and mortality. Fourth, hypochloremia has been confirmed to be associated with higher mortality in patients with heart failure; therefore, we additionally adjusted the history of congestive heart failure. Fifth, potassium-sparing diuretics and nonpotassium-sparing diuretics may have different effects on serum chloride concentration and outcomes [17], so we separately adjusted these 2 types of diuretics. Sixth, we used the multiple imputation method to impute all missing covariates to test the influence of these missing variables. Finally, to explore the potential influence of unmeasured confounders on our risk estimates, we performed an E-value analysis, which quantifies the required magnitude of an unmeasured confounder that would negate the observed association between serum chloride and mortality [32].

All statistical analyses were performed using the R program (version 4.2.1; R Foundation for Statistical Computing [33]) with package “survey” (version 4.1-1) for analysis of complex survey samples, package “survminer” (version 0.4.9) for survival curves plotting, package “rms” (version 6.6-0) for restricted cubic spline analysis, package “mice” (version 3.15.0) for multiple imputation, and package “Evalue” (version 4.1.3) for sensitivity analyses for unmeasured confounding and other biases. A 2-sided *P*<.05 was considered statistically significant.

## Results

### Selection of Participants

**Figure 1:**
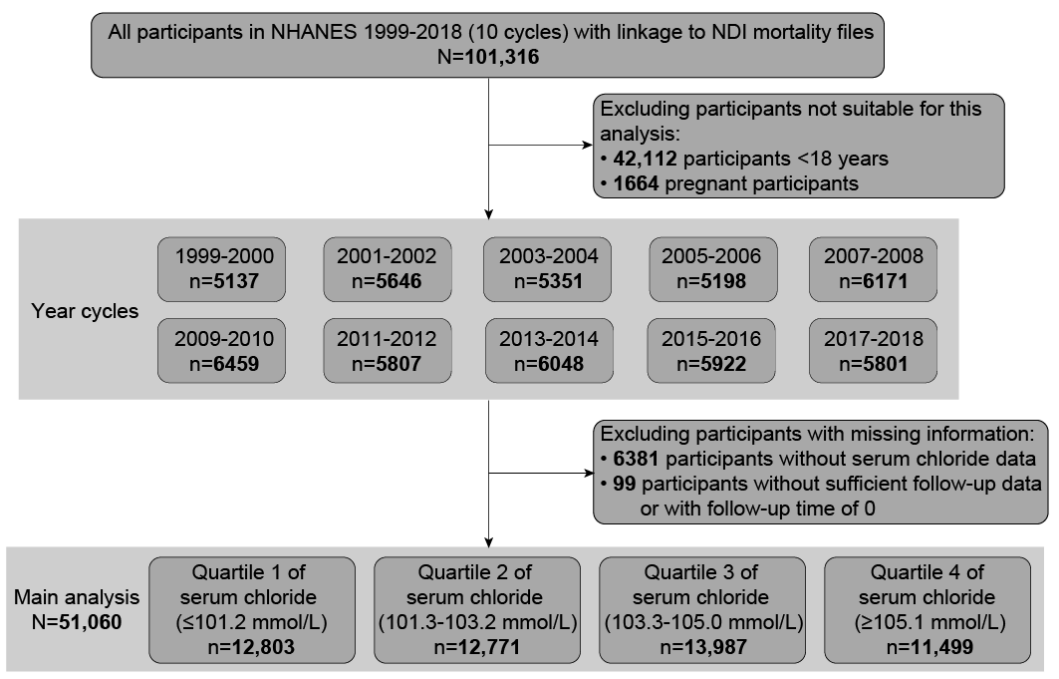
Flowchart of participant selection from the US National Health and Nutrition Examination Survey (NHANES) 1999-2018. NDI: National Death Index.

Of the total 101,316 participants in 10 cycles of NHANES (1999-2018), we excluded 42,112 participants aged <18 years, 1664 pregnant participants, 6381 participants without serum chloride data, and 99 participants without eligible follow-up data. The remaining 51,060 eligible participants were included in the final analysis (Figure 1).

### Baseline Characteristics of Participants

Among the 51,060 participants, representing 209 million American adults, 25,632 (50.95%) were female; mean age was 46.4 (SD 16.6) years; and 22,199 (68.48%) were non-Hispanic White. The median serum chloride of the participants was 103.2 (IQR 101.2-105.0) mmol/L, and according to the quartiles, serum chloride values (mmol/L) were categorized into 4 groups: quartile 1 (Q1; ≤101.2), quartile 2 (Q2; 101.3-103.2), quartile 3 (Q3; 103.3-105.0), and quartile 4 (Q4; ≥105.1).

During a median follow-up time of 111 (IQR 59-169) months, overall 7582 deaths were documented, of which 1958 (24.76%) were attributed to diseases of the heart, 1639 (22.72%) were attributed to malignant neoplasms, 430 (5.02%) were attributed to cerebrovascular diseases, 409 (6.16%) were attributed to chronic lower respiratory diseases, and 158 (1.85%) were attributed to influenza and pneumonia, and the leading causes of death were reorganized into CVD, cancer, and respiratory disease in the following analyses (Multimedia Appendix 1).

The population baseline characteristic profiles were compared across the quartiles of serum chloride (Table 1). Briefly, individuals with lower serum chloride were more likely to be older, male, non-Hispanic White, and nonmarried; to have higher HEI-2015, lower serum sodium, higher serum bicarbonate, lower eGFR, more use of diuretics; and to have a higher prevalence of hypertension, diabetes, CHD, stroke, COPD, and cancer (Table 1).

**Table 1:** Baseline characteristics of participants in different serum chloride quartiles from the US National Health and Nutrition Examination Survey 1999-2018 (N=51,060).

		Total	Q1^a^ (≤101.2)	Q2^b^ (101.3-103.2)	Q3^c^ (103.3-105.0)	Q4^d^ (≥105.1)	*P* value	
Sample size, n		51,060	12,803	12,771	13,987	11,499	N/A^e^	
**Sex, n (%)**								<.001
	Female	25,632 (50.95)	5722 (45.94)	6006 (48.16)	7337 (53.06)	6567 (57.48)		
	Male	25,428 (49.05)	7081 (54.06)	6765 (51.84)	6650 (46.94)	4932 (42.52)		
Age (years), mean (SD)		46.4 (16.6)	49.1 (17.2)	45.7 (16.4)	45.4 (16.3)	45.5 (16.1)	<.001	
**Race, n (%)**								<.001
	Black	10,619 (10.7)	2463 (9.6)	2590 (10.11)	2906 (10.79)	2660 (12.59)		
	Mexican	9388 (8.32)	2170 (7.11)	2318 (8.07)	2689 (8.87)	2211 (9.36)		
	White	22,199 (68.48)	5776 (70.01)	5601 (68.86)	6041 (68.64)	4781 (66.04)		
	Other	8854 (12.49)	2394 (13.28)	2262 (12.97)	2351 (11.7)	1847 (12.02)		
**Education, n (%)**								.003
	Below high school	12,867 (16.71)	3335 (17.9)	3057 (16.26)	3441 (16.74)	3034 (18.53)		
	High school or equivalent	10,994 (23.23)	2785 (24.9)	2706 (23.83)	2941 (23.51)	2562 (23.97)		
	College or above	23,630 (56.67)	5878 (57.19)	6031 (59.92)	6578 (59.75)	5143 (57.5)		
**Marital status, n (%)**								<.001
	Never married	9906 (17.95)	2433 (18.69)	2638 (19.42)	2716 (18.06)	2119 (17.79)		
	Married	24,924 (53.92)	6124 (53.74)	6212 (55.81)	6989 (57.07)	5599 (55.66)		
	Other	14,210 (25.11)	3825 (27.57)	3414 (24.77)	3736 (24.87)	3235 (26.55)		
Family income-to-poverty ratio, mean (SD)		2.98 (1.63)	2.97 (1.63)	3.04 (1.63)	3.01 (1.63)	2.89 (1.64)	<.001	
BMI (kg/m^2^), mean (SD)		28.7 (6.5)	28.6 (6.6)	28.3 (6.2)	28.5 (6.4)	29.3 (6.7)	<.001	
**Smoker, n (%)**								<.001
	Never	26,271 (52.57)	6446 (52.63)	6683 (54.72)	7291 (55.03)	5851 (52.76)		
	Former	11,903 (24.14)	3265 (26.15)	2928 (25.14)	3154 (23.86)	2556 (23.63)		
	Now	10,110 (20.93)	2501 (21.22)	2396 (20.15)	2723 (21.1)	2490 (23.62)		
**Alcohol user, n (%)**								.001
	Never	6538 (10.47)	1598 (11.42)	1612 (11.27)	1751 (11.64)	1577 (12.59)		
	Former	7600 (12.87)	1813 (13.99)	1750 (13.14)	2139 (14.62)	1898 (15.94)		
	Mild	14,440 (31.93)	3690 (36.19)	3771 (36.99)	3925 (35.72)	3054 (33.35)		
	Moderate	6464 (15.15)	1497 (16.13)	1633 (17.34)	1849 (17.29)	1485 (16.84)		
	Heavy	8742 (19.14)	2230 (22.26)	2188 (21.26)	2351 (20.73)	1973 (21.28)		
Healthy Eating Index-2015, mean (SD)		50.2 (13.6)	51.2 (14.0)	50.4 (13.9)	50.0 (13.5)	49.0 (13.0)	<.001	
Physical activity (metabolic equivalent tasks min/wk), mean (SD)		3423 (5638.7)	3359.1 (5659.6)	3466.5 (5812.3)	3315.3 (5476.3)	3581.7 (5604.0)	.07	
Serum sodium (mmol/L), mean (SD)		139.3 (2.3)	137.8 (2.5)	139.1 (2.0)	139.7 (1.9)	140.6 (1.9)	<.001	
Serum potassium (mmol/L), mean (SD)		4.01 (0.33)	4.00 (0.36)	4.01 (0.32)	4.01 (0.32)	4.03 (0.31)	.009	
Serum bicarbonate (mmol/L), mean (SD)		24.8(2.3)	25.5 (2.3)	25.1 (2.2)	24.7 (2.1)	23.9 (2.1)	<.001	
Estimated glomerular filtration rate (mL/min/1.73 m^2^), mean (SD)		94.7 (20.8)	92.6 (21.2)	94.9 (20.3)	95.8 (20.4)	95.4 (21.4)	<.001	
**Diuretics use, n (%)**								<.001
	No	44,379 (88.83)	9941 (80.45)	11,188 (89.51)	12,692 (92.54)	10,558 (93.47)		
	Yes	6632 (11.1)	2838 (19.55)	1573 (10.49)	1290 (7.46)	931 (6.53)		
**Hypertension, n (%)**								<.001
	No	30,501 (63.73)	6380 (54.28)	7791 (64.88)	9003 (68.28)	7327 (67.77)		
	Yes	20,545 (36.25)	6416 (45.72)	4977 (35.12)	4983 (31.72)	4169 (32.23)		
**Diabetes, n (%)**								<.001
	No	42,533 (87.34)	9591 (81.06)	10,773 (88.11)	12,118 (89.91)	10,051 (90.54)		
	Yes	8523 (12.66)	3210 (18.94)	1998 (11.89)	1869 (10.09)	1446 (9.46)		
**Coronary heart disease** **, n (%)**								<.001
	No	45,305 (93.01)	11,280 (95.32)	11,285 (96.65)	12,451 (97.06)	10,289 (96.77)		
	Yes	2040 (3.43)	674 (4.68)	478 (3.35)	466 (2.94)	422 (3.23)		
**Stroke, n (%)**								<.001
	No	45,663 (93.89)	11,440 (96.58)	11,381 (97.39)	12,506 (97.47)	10,336 (97.25)		
	Yes	1837 (2.73)	557 (3.42)	420 (2.61)	461 (2.53)	399 (2.75)		
**Chronic obstructive pulmonary disease** **, n (%)**								<.001
	No	45,581 (92.92)	11,347 (95.19)	11,383 (96.37)	12,494 (96.24)	10,357 (96.32)		
	Yes	2009 (3.85)	672 (4.81)	436 (3.63)	491 (3.76)	410 (3.68)		
**Cancer, n (%)**								<.001
	No	43,107 (87.48)	10,702 (89.05)	10,787 (91.35)	11,805 (90.72)	9813 (91.13)		
	Yes	4404 (9.14)	1310 (11.05)	1016 (8.65)	1154 (9.28)	924 (8.87)		

^a^Q1: quartile 1.

^b^Q2: quartile 2.

^c^Q3: quartile 3.

^d^Q4: quartile 4.

^e^N/A: not applicable.

### Associations of Serum Chloride Quartiles With All-Cause and Cause-Specific Mortality

**Figure 2:**
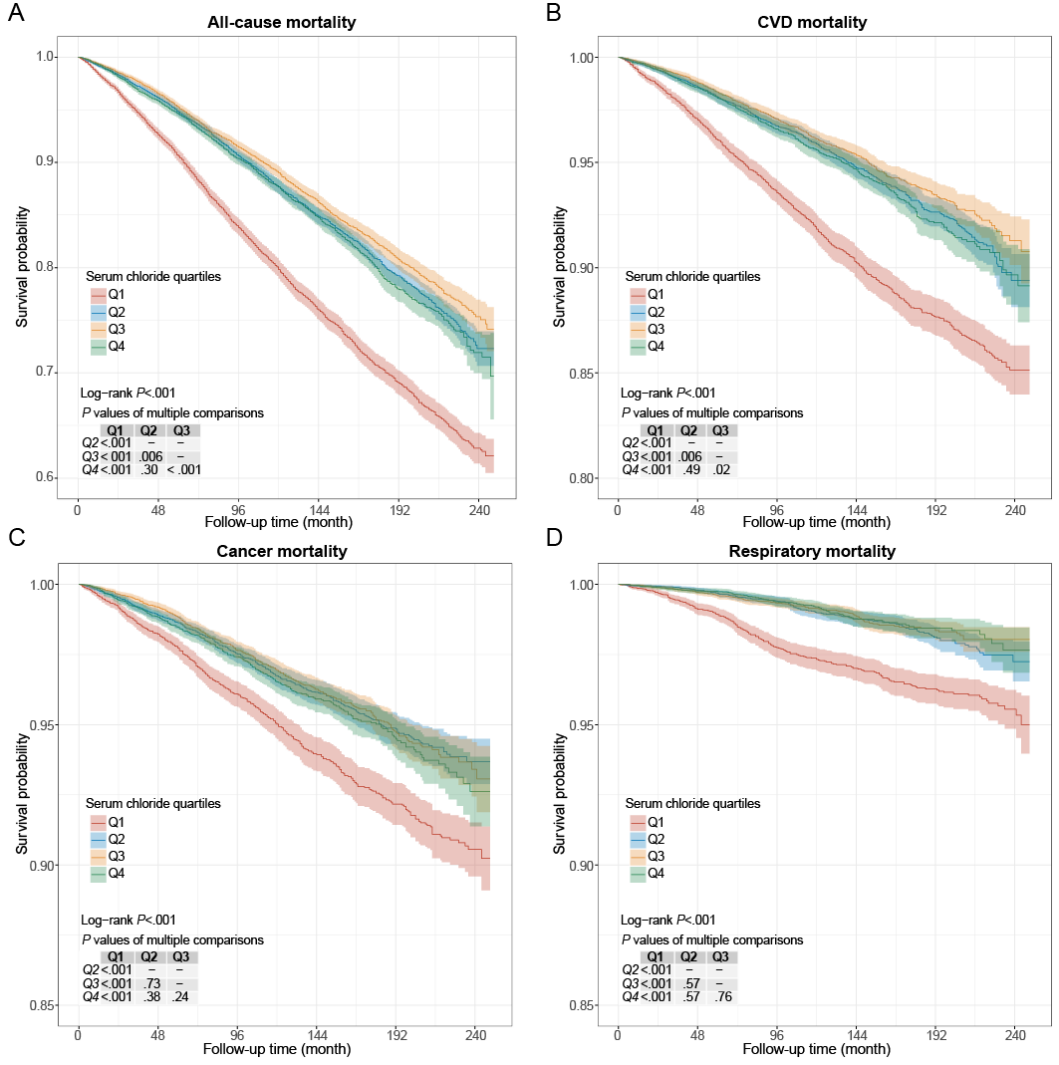
Survey-weighted cumulative hazard curves and log-rank tests comparing mortality risks owing to all-cause (A), cardiovascular disease (CVD; B), cancer (C), and respiratory disease (D) for individuals in different serum chloride quartiles from the US National Health and Nutrition Examination Survey 1999-2018. Q1: quartile 1; Q2: quartile 2; Q3: quartile 3; Q4: quartile 4.

The unadjusted survey-weighted Kaplan-Meier survival analyses, followed by the log-rank test and pairwise comparisons between groups with corrections, showed that individuals with the lowest quartiles of serum chloride consistently bear the highest mortality risk (Figure 2).

After multivariable adjustment, the all-cause mortality risk was lower in the Q2-Q4 group of serum chloride than in the Q1 group. Briefly, compared with the reference group (Q1), the HRs for all-cause mortality were estimated to be 0.77 (95% CI 0.67-0.89) for the Q2 group, 0.72 (95% CI 0.63-0.82) for the Q3 group, and 0.77 (95% CI 0.65-0.90) for the Q4 group (*P* for linear trend<.001; Table 2). As a continuous linear variable, every millimole per liter increment in serum chloride was associated with a 4% reduced risk of all-cause mortality (HR 0.96, 95% CI 0.94-0.98; Multimedia Appendix 2).

**Table 2:** Survey-weighted multivariate analyses of the associations of categorical serum chloride serum chloride with all-cause and cause-specific mortality for adults from the US National Health and Nutrition Examination Survey 1999-2018 (N=51,060)^a^.

		Q1^b^ (≤101.2)	Q2^c^ (101.3-103.2)			Q3^d^ (103.3-105.0)				Q4^e^ (≥105.1)			*P* value for trend^f^
		HR^g^ (95% CI)	HR (95% CI)	*P* value	HR (95% CI)		*P* value	HR (95% CI)			*P* value		
**All-cause mortality**													
	Crude	1.00 (reference)	0.58 (0.53-0.63)	<.001	0.51 (0.47-0.56)		<.001		0.59 (0.53-0.65)		<.001	<.001	
	Model 1^h^	1.00 (reference)	0.71 (0.65-0.76)	<.001	0.65 (0.60-0.70)		<.001		0.73 (0.67-0.79)		<.001	<.001	
	Model 2^i^	1.00 (reference)	0.73 (0.65-0.82)	<.001	0.67 (0.59-0.75)		<.001		0.72 (0.63-0.81)		<.001	<.001	
	Model 3^j^	1.00 (reference)	0.77 (0.67-0.89)	<.001	0.72 (0.63-0.82)		<.001		0.77 (0.65-0.90)		.001	<.001	
**Cardiovascular disease mortality**													
	Crude	1.00 (reference)	0.54 (0.47-0.62)	<.001	0.45 (0.39-0.53)		<.001		0.55 (0.46-0.65)		<.001	<.001	
	Model 1	1.00 (reference)	0.65 (0.58-0.74)	<.001	0.57 (0.49-0.66)		<.001		0.68 (0.58-0.80)		<.001	<.001	
	Model 2	1.00 (reference)	0.60 (0.50-0.72)	<.001	0.53 (0.42-0.66)		<.001		0.65 (0.53-0.81)		<.001	<.001	
	Model 3	1.00 (reference)	0.63 (0.51-0.79)	<.001	0.56 (0.43-0.73)		<.001		0.67 (0.50-0.89)		.006	.004	
**Cancer mortality**													
	Crude	1.00 (reference)	0.55 (0.46-0.66)	<.001	0.58 (0.49-0.70)		<.001		0.63 (0.51-0.76)		<.001	<.001	
	Model 1	1.00 (reference)	0.64 (0.53-0.76)	<.001	0.67 (0.56-0.81)		<.001		0.75 (0.62-0.90)		.003	.002	
	Model 2	1.00 (reference)	0.69 (0.54-0.86)	.001	0.67 (0.52-0.86)		.002		0.72 (0.58-0.91)		.005	.005	
	Model 3	1.00 (reference)	0.67 (0.54-0.84)	<.001	0.65 (0.50-0.85)		.002		0.65 (0.48-0.87)		.004	.004	
**Respiratory mortality**													
	Crude	1.00 (reference)	0.44 (0.34-0.57)	<.001	0.40 (0.31-0.51)		<.001		0.38 (0.28-0.50)		<.001	<.001	
	Model 1	1.00 (reference)	0.54 (0.41-0.70)	<.001	0.49 (0.39-0.62)		<.001		0.47 (0.36-0.62)		<.001	<.001	
	Model 2	1.00 (reference)	0.63 (0.39-1.02)	.06	0.57 (0.39-0.82)		.003		0.50 (0.32-0.81)		.004	.001	
	Model 3	1.00 (reference)	0.68 (0.41-1.13)	.14	0.59 (0.40-0.88)		.009		0.51 (0.31-0.84)		.008	.004	

^a^Data were fitted to a multivariate Cox proportional hazards model for data from a complex survey design.

^b^Q1: quartile 1.

^c^Q2: quartile 2.

^d^Q3: quartile 3.

^e^Q4: quartile 4.

^f^The test for trend was based on the variable containing the median value for each quartile.

^g^HR: hazard ratio.

^h^Model 1: adjusted for sex, age, and race.

^i^Model 2: adjusted for sex, age, race, education, marital status, family income-to-poverty ratio, BMI, smoking, alcohol use, Healthy Eating Index-2015, and physical activity.

^j^Model 3: adjusted for sex; age; race; education; marital status; family income-to-poverty ratio; BMI; smoking; alcohol use; Healthy Eating Index-2015; physical activity; serum sodium; serum potassium; serum bicarbonate; estimated glomerular filtration rate; use of diuretics; and comorbidity or history of hypertension, diabetes, coronary heart disease, stroke, chronic obstructive pulmonary disease, and cancer.

Similarly, CVD mortality was consistently lower in the Q2-Q4 group of serum chloride than in the Q1 group in all models. After being fully adjusted, the HRs for CVD mortality were estimated to be 0.63 (95% CI 0.51-0.79) for the Q2 group, 0.56 (95% CI 0.43-0.73) for the Q3 group, and 0.67 (95% CI 0.50-0.89) for the Q4 group compared with the Q1 group (*P* for linear trend=.004; Table 2). As a continuous linear variable, every millimole per liter increment in serum chloride was associated with a 7% reduced risk of CVD mortality (HR 0.93, 95% CI 0.89-0.97; Multimedia Appendix 2).

Moreover, cancer mortality was consistently lower in the Q2-Q4 group of serum chloride than in the Q1 group. With the total adjustment, the HRs for cancer mortality were estimated to be 0.67 (95% CI 0.54-0.84) for the Q2 group, 0.65 (95% CI 0.50-0.85) for the Q3 group, and 0.65 (95% CI 0.48-0.87) for the Q4 group compared with the Q1 group (*P* for linear trend=.004; Table 2). As a continuous linear variable, every millimole per liter increment in serum chloride was associated with a 6% reduced risk of cancer disease mortality (HR 0.94, 95% CI 0.89-0.98; Multimedia Appendix 2).

Similarly, consistently lower respiratory mortality was observed in Q3 and Q4 groups than in the Q1 group. With the full adjustment, the HRs for respiratory mortality were estimated to be 0.68 (95% CI 0.41-1.13) for the Q2 group, 0.59 (95% CI 0.40-0.88) for the Q3 group, and 0.51 (95% CI 0.31-0.84) for the Q4 group compared with the Q1 group (*P* for linear trend=.004; Table 2), and every millimole per liter increment in serum chloride was associated with a 15% reduced risk of respiratory mortality (HR 0.85, 95% CI 0.80-0.92; Multimedia Appendix 2).

### The Nonlinear Analyses of the Association Between Continuous Serum Chloride and Mortality

**Figure 3:**
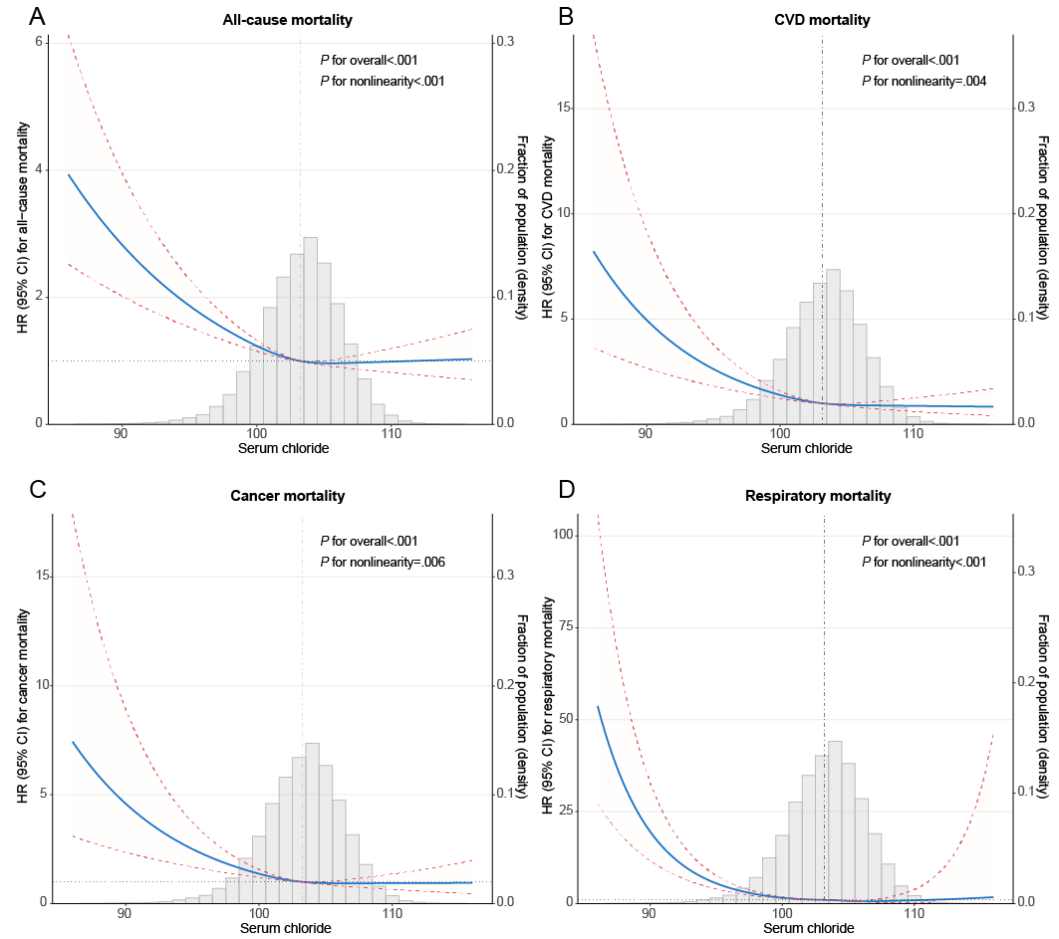
Survey-weighted restricted cubic spline analyses of the associations of continuous serum chloride with all-cause mortality (A), cardiovascular disease (CVD) mortality (B), cancer mortality (C), and respiratory mortality (D), and the probability distribution histogram is represented in the background. All models were adjusted for sex; age; race; education; marital status; family income-to-poverty ratio; BMI; smoking status; alcohol use; Healthy Eating Index-2015; physical activity; serum sodium; serum potassium; serum bicarbonate; estimated glomerular filtration rate; use of diuretics; and comorbidity or history of hypertension, diabetes, coronary heart disease, stroke, chronic obstructive pulmonary disease, and cancer. Solid blue lines are multivariable-adjusted HR estimations, and the dashed red lines are the corresponding 95% CIs. The reference point was set at the median (103.2 mmol/L). HR: hazard ratio.

Spline models with fully adjusted covariates were constructed to profile a more direct relationship between serum chloride and mortality. An L-shaped association was observed between serum chloride and all-cause mortality (nonlinear *P*<.001), in which all-cause mortality risk decreased steeply until around the median of serum chloride and then tended to be stable (Figure 3A). Similarly, L-shaped associations were observed between serum chloride and CVD mortality (nonlinear *P*=.004; Figure 3B), cancer mortality (nonlinear *P*=.006; Figure 3C), and respiratory mortality (nonlinear *P*<.001; Figure 3D).

### Subgroup Analyses of the Association of Serum Chloride With Mortality

Subgroup analyses were performed to depict the potential different associations of serum chloride quartiles and mortality in subpopulations. For the association with the primary outcome, all-cause mortality, no significant interactions were found between serum chloride and all these stratification variables (all *P* for interaction>.05), implying similar associations of serum chloride with all-cause mortality were detected in these subgroups (Table 3). Similarly, consistent associations of serum chloride with CVD mortality were detected across different subpopulations, and no significant interactions were detected (all *P* for interaction>.05; Multimedia Appendix 3). Regarding cancer mortality, more prominent associations of low chloride with mortality risk were detected in smokers and participants with hypertension, as shown by the interaction effect of serum chloride with smoking status (*P* for interaction=.04) and comorbid hypertension (*P* for interaction=.047; Multimedia Appendix 4). With regard to respiratory mortality, more prominent associations of low chloride with mortality risk were detected in adults with less physical activity, as indicated by its potential interaction effect with serum chloride (*P* for interaction=.02; Multimedia Appendix 5).

**Table 3:** Subgroup analyses of the associations of serum chloride quartiles with all-cause mortality for adults from the US National Health and Nutrition Examination Survey 1999-2018 (N=51,060)^a^.

		Sample size, n	Q1^b^ (≤101.2)	Q2^c^ (101.3-103.2)	Q3^d^ (103.3-105.0)	Q4^e^ (≥105.1)	*P* value for trend	*P* value for interaction	
			HR^f^ (95% CI)	HR (95% CI)	HR (95% CI)	HR (95% CI)			
**Sex**									.66
	Female	25,632	1.00 (reference)	0.77 (0.63-0.95)	0.80 (0.64-1.00)	0.83 (0.65-1.05)	.09		
	Male	25,428	1.00 (reference)	0.76 (0.63-0.91)	0.66 (0.55-0.79)	0.74 (0.60-0.91)	.002		
**Age (years)**									.59
	≤60	35,508	1.00 (reference)	0.81 (0.63-1.06)	0.78 (0.61-1.00)	0.77 (0.56-1.07)	.09		
	>60	15,552	1.00 (reference)	0.71 (0.61-0.82)	0.69 (0.59-0.82)	0.78 (0.64-0.95)	.004		
**Race**									.25
	White	22,199	1.00 (reference)	0.77 (0.66-0.89)	0.69 (0.58-0.81)	0.76 (0.63-0.92)	.002		
	People of racial and ethnic minorities	28,861	1.00 (reference)	0.81 (0.64-1.03)	0.87 (0.70-1.08)	0.78 (0.59-1.04)	.09		
**Education**									.87
	High school or below	23,861	1.00 (reference)	0.75 (0.63-0.89)	0.67 (0.55-0.80)	0.71 (0.59-0.86)	<.001		
	College or above	23,630	1.00 (reference)	0.82 (0.67-1.00)	0.81 (0.66-1.00)	0.86 (0.66-1.13)	.22		
**Marital status**									.29
	Married	24,924	1.00 (reference)	0.81 (0.69-0.96)	0.75 (0.64-0.88)	0.73 (0.60-0.89)	<.001		
	Other	24,116	1.00 (reference)	0.73 (0.59-0.91)	0.69 (0.56-0.85)	0.85 (0.65-1.10)	.13		
**Family income-to-poverty ratio**									.19
	≤1.3	14,912	1.00 (reference)	0.74 (0.58-0.94)	0.73 (0.59-0.90)	0.83 (0.63-1.09)	.18		
	>1.3	31,676	1.00 (reference)	0.80 (0.68-0.94)	0.72 (0.60-0.85)	0.73 (0.60-0.89)	<.001		
**BMI**									.65
	≤28	26,350	1.00 (reference)	0.75 (0.65-0.87)	0.68 (0.56-0.81)	0.75 (0.61-0.91)	.001		
	>28	23,827	1.00 (reference)	0.80 (0.64-0.98)	0.77 (0.63-0.94)	0.80 (0.62-1.03)	.07		
**Smoker**									.11
	Nonsmoker	26,271	1.00 (reference)	0.79 (0.65-0.96)	0.84 (0.68-1.04)	1.05 (0.81-1.35)	.90		
	Smoker	22,013	1.00 (reference)	0.75 (0.63-0.89)	0.65 (0.53-0.79)	0.65 (0.53-0.79)	<.001		
**Current drinker**									.97
	No	14,138	1.00 (reference)	0.78 (0.63-0.96)	0.71 (0.57-0.88)	0.78 (0.60-1.02)	.047		
	Yes	29,646	1.00 (reference)	0.78 (0.66-0.93)	0.74 (0.63-0.88)	0.78 (0.64-0.96)	.007		
**Healthy Eating Index** **-2015**									.14
	≤50	24,430	1.00 (reference)	0.69 (0.57-0.82)	0.66 (0.54-0.80)	0.75 (0.60-0.94)	.006		
	>50	23,604	1.00 (reference)	0.88 (0.74-1.04)	0.79 (0.65-0.95)	0.80 (0.66-0.98)	.02		
**Physical activity**									.95
	Inactive	13,114	1.00 (reference)	0.80 (0.67-0.95)	0.77 (0.63-0.93)	0.87 (0.69-1.09)	.10		
	Active	23,689	1.00 (reference)	0.76 (0.62-0.92)	0.67 (0.56-0.81)	0.70 (0.55-0.90)	.003		
**Diuretics use**									.14
	No	44,379	1.00 (reference)	0.81 (0.69-0.95)	0.72 (0.61-0.85)	0.75 (0.63-0.89)	<.001		
	Yes	6632	1.00 (reference)	0.67 (0.52-0.85)	0.73 (0.58-0.93)	0.90 (0.66-1.24)	.09		
**Hypertension**									.46
	No	30,501	1.00 (reference)	0.88 (0.69-1.12)	0.85 (0.66-1.10)	0.96 (0.71-1.29)	.74		
	Yes	20,545	1.00 (reference)	0.72 (0.62-0.83)	0.65 (0.55-0.76)	0.68 (0.57-0.81)	<.001		
**Diabetes**									.32
	No	42,533	1.00 (reference)	0.81 (0.69-0.94)	0.71 (0.61-0.83)	0.76 (0.63-0.91)	.002		
	Yes	8523	1.00 (reference)	0.66 (0.52-0.84)	0.73 (0.57-0.93)	0.80 (0.59-1.09)	.04		
**Coronary heart disease**									.48
	No	45,305	1.00 (reference)	0.76 (0.66-0.88)	0.71 (0.62-0.82)	0.78 (0.66-0.92)	.001		
	Yes	2040	1.00 (reference)	0.89 (0.67-1.18)	0.73 (0.50-1.08)	0.72 (0.47-1.10)	.11		
**Stroke**									.76
	No	45,663	1.00 (reference)	0.78 (0.67-0.90)	0.71 (0.62-0.82)	0.76 (0.64-0.90)	<.001		
	Yes	1837	1.00 (reference)	0.65 (0.40-1.07)	0.87 (0.51-1.46)	0.91 (0.52-1.60)	.66		
**Chronic obstructive pulmonary disease**									.69
	No	45,581	1.00 (reference)	0.79 (0.68-0.92)	0.73 (0.62-0.84)	0.77 (0.65-0.91)	.001		
	Yes	2009	1.00 (reference)	0.69 (0.45-1.07)	0.72 (0.47-1.10)	0.84 (0.48-1.46)	.42		
**Cancer**									.54
	No	43,107	1.00 (reference)	0.83 (0.72-0.96)	0.78 (0.67-0.91)	0.85 (0.71-1.03)	.053		
	Yes	4404	1.00 (reference)	0.57 (0.42-0.76)	0.52 (0.39-0.68)	0.51 (0.36-0.73)	<.001		
**C** **hronic kidney disease**									.19
	No	41,298	1.00 (reference)	0.84 (0.71-1.00)	0.77 (0.65-0.92)	0.79 (0.64-0.96)	.01		
	Yes	9215	1.00 (reference)	0.68 (0.57-0.82)	0.63 (0.52-0.76)	0.72 (0.56-0.94)	.007		

^a^Data were calculated using svycoxph to fit a multivariate Cox proportional hazards model to data from a complex survey design, with adjustment of dichotomic sex; age; race; education; marital status; family income-to-poverty ratio; BMI; smoking; alcohol use; Healthy Eating Index-2015; physical activity; serum sodium; serum potassium; serum bicarbonate; use of diuretics; and comorbidity or history of hypertension, diabetes, coronary heart disease, stroke, chronic obstructive pulmonary disease, cancer, and chronic kidney disease, except for the specific stratification variable. The test for trend was based on the variable containing the median value for each quartile. The interaction effect was evaluated using the likelihood ratio test.

^b^Q1: quartile 1.

^c^Q2: quartile 2.

^d^Q3: quartile 3.

^e^Q4: quartile 4.

^f^HR: hazard ratio.

### Sensitivity Analyses of the Association of Serum Chloride With Mortality

All sensitivity analyses showed a robust association of serum chloride with all-cause mortality and cause-specific mortality. Briefly, after excluding participants who died in the first 2 years (992/51,060, 1.94%), the association of categorical serum chloride with all-cause and cause-specific mortality remained stable (Multimedia Appendix 6). The association of categorical serum chloride with mortality was slightly attenuated in the fully adjusted model, especially with cancer mortality, when excluding participants with potential serum chloride outliers (936/51,060, 1.83%; Multimedia Appendix 7). In addition, excluding participants with possible hypoalbuminemia (714/51,060, 1.4%) did not cause substantial shifts in the results (Multimedia Appendix 8). Additional adjustment of history of congestive heart failure or separate adjustment of potassium-sparing diuretics and nonpotassium-sparing diuretics did not cause substantial shifts in the results (Multimedia Appendix 9). Furthermore, there were some missing data on covariates (Multimedia Appendix 10), and multiple imputation of them strengthened the associations of serum chloride with all-cause and cause-specific mortality, probably owing to the increased power (Multimedia Appendix 11).

Finally, we calculated E-values to assess the sensitivity to unmeasured confounding. Briefly, the all-cause mortality risk of low serum chloride (Q1) could be influenced only when the unmeasured covariates had risk ratios >1.92 for both serum chloride and all-cause mortality, and similarly, to account for the association we observed between serum chloride and cause-specific mortality and to address any unmeasured confounding, the strength of the relationship would need to be >2.34, 2.35, and 2.78 (Multimedia Appendix 12).

## Discussion

### Principal Findings

In this study, we used a prospective cohort with a relatively large sample size to explore the link between serum chloride level and all-cause and cause-specific mortality in American adults. After adjusting for demographic factors, relevant lifestyle, and comorbid factors, low serum chloride levels were significantly associated with higher risks of all-cause mortality, CVD mortality, cancer mortality, and respiratory disease mortality within a certain range. Further analysis revealed an L-shaped relationship between serum chloride levels and all-cause and specific-cause mortality in the general population. The associations of serum chloride and mortality risk were consistent across different subgroup populations and were rather robust in different settings. These findings suggested that serum chloride may serve as a promising cost-effective health indicator in the general adult population.

The widely acknowledged normal range of serum chloride for an adult is 96 to 106 mmol/L [2], and in our study population, the serum chloride was 103.2 (101.2-105.0) mmol/L, which subtly fluctuated according to sex, age, or race as well as lifestyle factors and clinical situations. We found that participants with lower serum chloride tended to be older and have a lower BMI. Considering that low chloride levels may be a symptom of malnutrition [34], lower BMI and older age might reflect a nutritional deficiency in the population. In addition, lower chloride levels are also observed in people with comorbidities such as hypertension, kidney disease, and diabetes mellitus.

The association of remarkable abnormality of serum chloride, hypochloremia, and hyperchloremia with mortality risk has been investigated in various hospital settings. Thus, serum chloride level (<100 mmol/L) in patients with hypertension was independently associated with a 20% higher mortality rate, as shown by a cohort of 12,968 patients with hypertension [35]. Similarly, serum chloride levels were found to be inversely associated with all-cause mortality in patients with CKD [14] and with intensive care unit mortality in patients with cirrhosis who were critically ill [36]. However, in the community-based population, the association between serum chloride distribution and mortality has been scarcely discussed and seems contradictory. Consistent with our study, a cohort study in the general population in Belgium that was followed up for 10 years showed that serum chloride levels <100 mmol/L were associated with increased all-cause, CVD, and non-CVD mortality [37]. Although we drew a conclusion similar to their study, we studied a different target population with a larger sample size and included different covariates. Another cohort study including 16,483 participants in NHANES III (1988-1994) followed up to 2011 found that low serum sodium level was associated with increased cardiovascular mortality, whereas lower serum chloride level was not, which was inconsistent with this study [38]. In our study, we included 51,060 participants (1999-2018) and followed them up to 2019. We included more covariates and conducted a more comprehensive analysis to confirm the results. Thus, the differences in the sample size, follow-up time, and statistical method might explain this discrepancy.

In our study, we found that low chloride level was not only associated with all-cause mortality but also with cause-specific mortality, including CVD mortality, cancer mortality, and respiratory mortality. Consistent with our findings, various studies have shown that lower chloride levels are associated with CVD mortality and play a prognostic role in CVD [11,37,39,40]. Several hypotheses have been proposed regarding the mechanism behind this. First, lower chloride levels or hypochloremia could increase the activity of a family of with no lysine kinases, which leads to diuretic resistance [41]. Second, as chloride, not sodium, is the principal regulator of renin release from the juxtaglomerular apparatus, hypochloremia can decrease chloride delivery to the macula densa and hence increase the renin release, which has long-term deleterious effects in patients with heart issues, such as venous congestion and pathologic myocardial remodeling [42]. Third, hypochloremia may impair the activity of the cardiac chloride channel, which may result in impaired cardiac contractility and arrhythmia and lead to sudden death [43,44]. Furthermore, electrolyte imbalance including hypochloremia can result in the dysregulation of myocyte intracellular pH, which also contributes to arrhythmia [45].

Studies on the association between serum chloride and cancer mortality are rare. A retrospective study in China demonstrated that low chloride levels were associated with increased mortality in patients with colorectal cancer after surgery [46]. Another prospective study in a hospital in Saudi Arabia reported that lower chloride level was a unique prognostic marker for high-risk polyps, which was associated with colorectal cancer [47]. Several hypotheses may elucidate the underlying mechanism. A cancer diagnosis may induce chronic stress in patients [48], which causes relative disruption of adrenal hormones and results in a decrease of chloride level [49]. Tumor-associated abnormal expression of some ion channel proteins can also lead to electrolyte disorders including hypochloremia and thus lead to adverse outcomes [50]. In addition, our subgroup analysis showed that smokers and adults with hypertension might be more susceptible to cancer mortality related to low serum chloride levels; however, further studies are needed to clarify the potential mechanisms.

Literature on the association between serum chloride and respiratory mortality is also limited. Consistent with our study, a retrospective cohort study in Glasgow showed that mortality in patients with COPD increased linearly as serum chloride levels <105 mmol/L [51]. Lower chloride level is a metabolic indicator of chronic respiratory acidosis [52]. Patients with COPD usually have an increase in partial pressure of carbon dioxide and bicarbonate; thus, chloride decrease may be a renal response to the increasing partial pressure of carbon dioxide to assure the electroneutrality. Moreover, our subgroup analysis indicated that participants with less physical activity may bear a higher respiratory mortality risk with low chloride compared with the counterparts with active physical activity, and further studies are needed to fully elucidate the mechanism.

Compared with Q1 and Q4 groups, participants in the Q2 and Q3 groups of serum chloride tend to have relative lower hazards, probably because their serum chloride levels (101.3-105.0 mmol/L) represents an ideal homeostasis which may, in some degree, avoid the risk of adverse events related to hypochloremia or hyperchloremia. Surprisingly, our study demonstrated that all-cause and specific-cause mortality did not increase when the serum chloride level was >105.1 mmol/L, which is in accordance with a recent community-based study conducted in Israel [53] and studies involving people with high blood pressure [35] or those with chronic heart problems [9]. However, some inconsistent results are also noteworthy, in which a retrospective cohort in South Korea showed that the 90-day mortality was increased in patients after noncardiac surgery when serum chloride level was >110 mmol/L [54], and early hyperchloremia was associated with higher odds of death or disability at 90-day, in patients with acute ischemic stroke [55], and high serum chloride level at admission was associated with higher in-hospital mortality in patients in medical intensive care units [56]. The significant difference in the clinical characteristics, demographics, and laboratory variables in the different population settings may partially explain this discrepancy. Moreover, previous literature suggests that hyperchloremia is often hospital acquired, whereas hypochloremia is community acquired [57], in which people with unfavorably high serum chloride levels or hyperchloremia are more likely to be patients in the hospital but not individuals from the community, as in our study.

Fluctuation of serum chloride usually occurs with concomitant changes in serum sodium, potassium, and bicarbonate; therefore, we adjusted them in the final models and confirmed the chloride-specific risk independent of hyponatremia or acid-base disturbances. Serum chloride is largely influenced by renal function and the use of diuretics; therefore, we adjusted eGFR and diuretic use in the final model and performed stratified analysis according to the renal function and diuretic use, validating the consistent association of low serum chloride with mortality risk. In our study, the cutoff point for low serum chloride was set as the first quartile of chloride distribution in the population (101.2 mmol/L), whereas hypochloremia was commonly defined as ≤96 mmol/L [10,58], and some researchers selected 100 mmol/L as the cutoff point [13,35]; therefore, the differences in definition criteria should be taken into account when explaining the effect size of low serum chloride on mortality risk.

This study had several strengths, including the relatively large sample size (>50,000) and the nationally representative sample design, which facilitates the generalization of our findings. Moreover, we took into account a multitude of potential confounding factors, including sociodemographic factors, lifestyle factors, laboratory tests, drug use, and comorbidities with extensive statistical methods, which guaranteed the robustness of our findings.

### Limitations

However, some limitations of this study must be acknowledged. First, causality cannot be determined owing to the observational study design, which requires randomized controlled studies for confirmation. Second, the serum chloride concentration was investigated only once at baseline, which may be influenced by temporary factors; therefore, repeated measurements are advisable in future studies. Third, the study participants were US adult civilians; thus, the associations and size effect need to be confirmed when trying to generalize the results to other populations. Finally, the possibility of residual and unknown confounding cannot be entirely excluded.

### Conclusions

In conclusion, we found that decreased serum chloride concentrations were independently associated with increased all-cause mortality, CVD mortality, cancer mortality, and respiratory disease mortality in the American adult population, and these findings emphasized the importance of monitoring serum chloride in health evaluation. However, further studies are still warranted to verify the predictive role of serum chloride and to explore the potential pathophysiological mechanisms underlying the differences in mortality.

## Appendix

Multimedia Appendix 1 The distribution of leading causes of death in the enrolled participants with survey-weighted percentages from the US National Health and Nutrition Examination Survey 1999-2018.

Multimedia Appendix 2 Survey-weighted multivariate analyses of the associations of continuous serum chloride with all-cause and cause-specific mortality for adults from the US National Health and Nutrition Examination Survey 1999-2018.

Multimedia Appendix 3 Subgroup analyses of the associations of serum chloride quartiles with cardiovascular disease mortality for adults from the US National Health and Nutrition Examination Survey 1999-2018.

Multimedia Appendix 4 Subgroup analyses of the associations of serum chloride quartiles with cancer mortality for adults from the US National Health and Nutrition Examination Survey 1999-2018.

Multimedia Appendix 5 Subgroup analyses of the associations of serum chloride quartiles with respiratory disease mortality for adults from the US National Health and Nutrition Examination Survey 1999-2018.

Multimedia Appendix 6 Survey-weighted multivariate analyses of the associations of categorical serum chloride with all-cause and cause-specific mortality after excluding participants who died in the first 2 years for adults from the US National Health and Nutrition Examination Survey 1999-2018.

Multimedia Appendix 7 Survey-weighted multivariate analyses of the associations of categorical serum chloride with all-cause and cause-specific mortality after excluding participants with potential serum chloride outliers for adults from the US National Health and Nutrition Examination Survey 1999-2018.

Multimedia Appendix 8 Survey-weighted multivariate analyses of the associations of categorical serum chloride with all-cause and cause-specific mortality after excluding participants with possible hypoalbuminemia for adults from the US National Health and Nutrition Examination Survey 1999-2018.

Multimedia Appendix 9 Survey-weighted multivariate analyses of the associations of categorical serum chloride with all-cause and cause-specific mortality after additionally adjusting the history of congestive heart failure and separately adjusted use of potassium-sparing diuretics and potassium-wasting diuretics for adults from the US National Health and Nutrition Examination Survey 1999-2018.

Multimedia Appendix 10 Summary of missing data in covariates in analyses from the US National Health and Nutrition Examination Survey 1999-2018.

Multimedia Appendix 11 Survey-weighted multivariate analyses of the associations of categorical serum chloride with all-cause and cause-specific mortality for adults after imputation all missing covariates from the US National Health and Nutrition Examination Survey 1999-2018.

Multimedia Appendix 12 E-value analysis for categorical serum chloride with all-cause and cause-specific mortality in adult participants from the US National Health and Nutrition Examination Survey 1999-2018.

## Abbreviations

CHD: coronary heart disease
CKD: chronic kidney disease
COPD: chronic obstructive pulmonary disease
CVD: cardiovascular disease
eGFR: estimated glomerular filtration rate
HR: hazard ratio
MET: metabolic equivalent task
NHANES: National Health and Nutrition Examination Survey
PIR: family income-to-poverty ratio
Q1: quartile 1
Q2: quartile 2
Q3: quartile 3
Q4: quartile 4
STROBE: Strengthening the Reporting of Observational Studies in Epidemiology

## Data Availability

The data sets were derived from National Health and Nutrition Examination Survey 1999-2018 [59], and only publicly available data were used in this study. The data sets generated or analyzed during this study are available from the corresponding author upon reasonable request.
