# Supplementary figures and images for: L-Shaped Association of Serum Chloride Level With All-Cause and Cause-Specific Mortality in American Adults: Population-Based Prospective Cohort Study
Source: JMIR Public Health Surveill. 2023 Nov 13;9:e49291. doi: 10.2196/49291 (PMC10682926; doi:10.2196/49291)

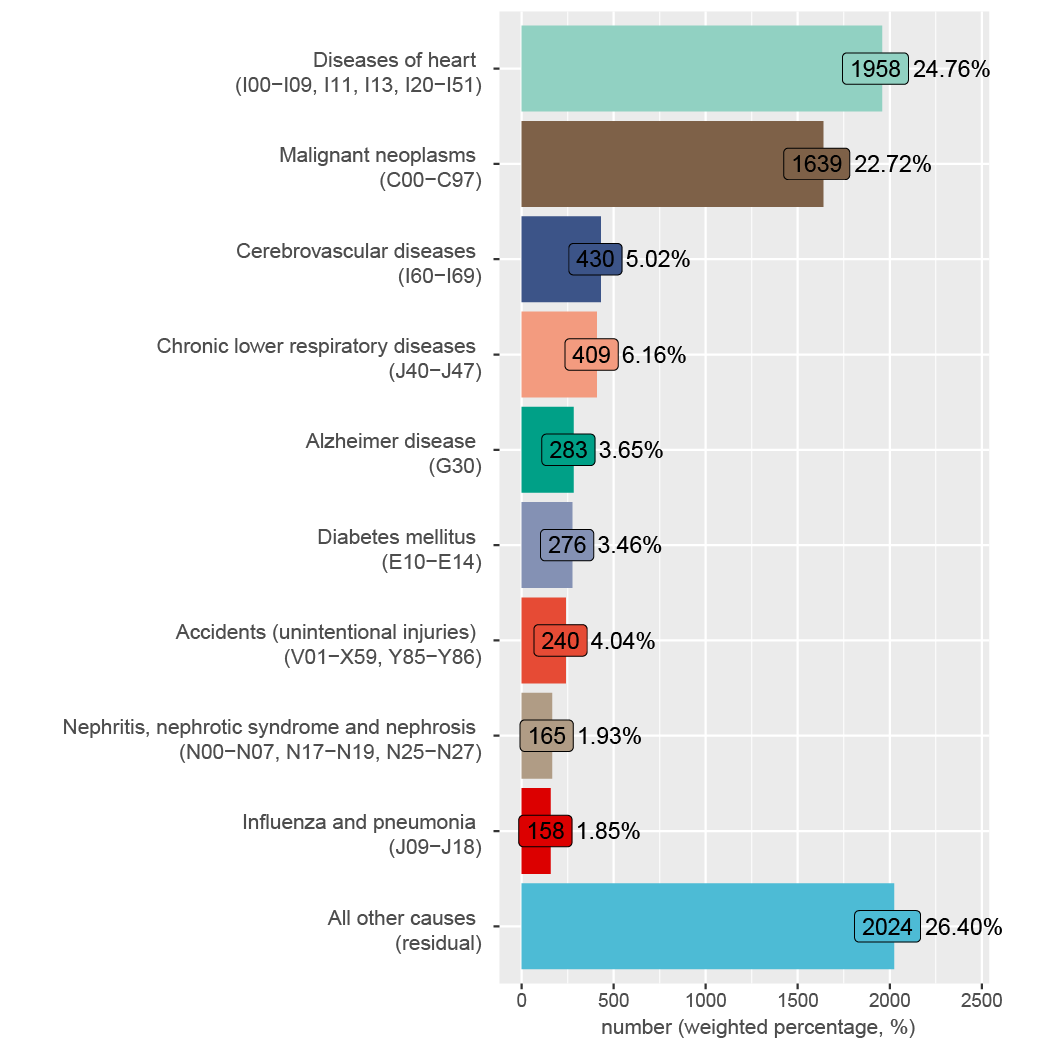

Supplement: Multimedia Appendix 1 [file publichealth_v9i1e49291_app1.png]

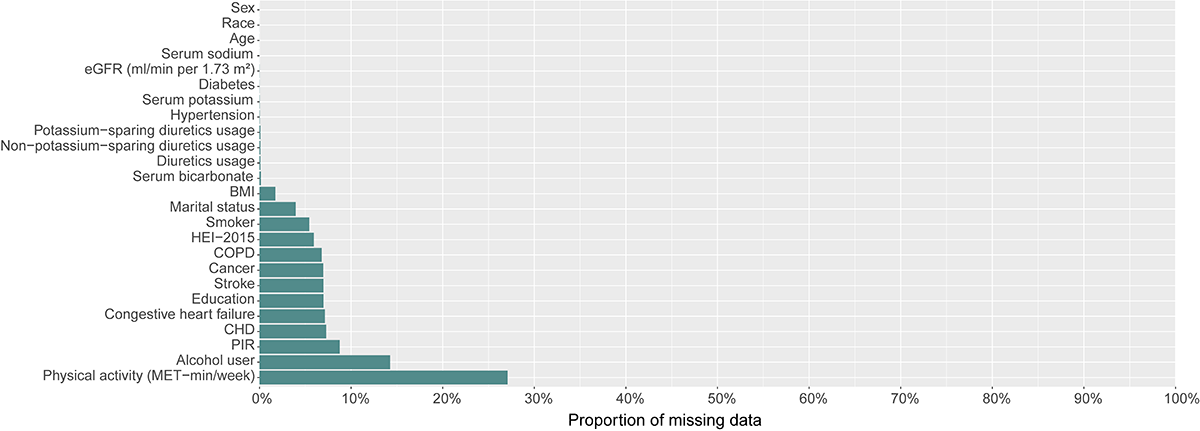

Supplement: Multimedia Appendix 10 [file publichealth_v9i1e49291_app10.png]
