# Supplementary material for: L-Shaped Association of Serum Chloride Level With All-Cause and Cause-Specific Mortality in American Adults: Population-Based Prospective Cohort Study
Source: JMIR Public Health Surveill. 2023 Nov 13;9:e49291. doi: 10.2196/49291 (PMC10682926; doi:10.2196/49291)
Supplement: Multimedia Appendix 2 [file publichealth_v9i1e49291_app2.doc]

| **Table S1. Survey-weighted multivariate analyses of the associations of continuous serum chloride with all-cause and cause-specific mortality for adults from the US National Health and Nutrition Examination Survey (NHANES) 1999-2018.** | | |
| --- | --- | --- |
|  | HR (95% CI) | P-value |
| **All-cause mortality** |  |  |
| **Crude** | 0.91(0.90,0.92) | <.001 |
| **Model 1** | 0.95(0.94,0.96) | <.001 |
| **Model 2** | 0.95(0.94,0.96) | <.001 |
| **Model 3** | 0.96(0.94,0.98) | <.001 |
| **CVD mortality** |  |  |
| **Crude** | 0.89(0.87,0.91) | <.001 |
| **Model 1** | 0.93(0.91,0.95) | <.001 |
| **Model 2** | 0.93(0.91,0.96) | <.001 |
| **Model 3** | 0.93(0.89,0.97) | <.001 |
| **Cancer mortality** |  |  |
| **Crude** | 0.92(0.89,0.95) | <.001 |
| **Model 1** | 0.95(0.93,0.98) | <.001 |
| **Model 2** | 0.95(0.92,0.99) | .006 |
| **Model 3** | 0.94(0.89,0.98) | .006 |
| **Respiratory mortality** |  |  |
| **Crude** | 0.85(0.83,0.87) | <.001 |
| **Model 1** | 0.89(0.87,0.92) | <.001 |
| **Model 2** | 0.90(0.86, 0.94) | <.001 |
| **Model 3** | 0.85(0.80, 0.92) | <.001 |

| Data were fitted to a multivariate Cox proportional hazards model for data from a complex survey design.  Model 1: Adjusted for sex, age, and race.  Model 2: Adjusted for sex, age, race, education, marital status, PIR, BMI, smoking, alcohol use, HEI-2015, and physical activity.  Model 3: Adjusted for sex, age, race, education, marital status, PIR, BMI, smoking, alcohol use, HEI-2015, physical activity, serum sodium, serum potassium, serum bicarbonate, eGFR, usage of diuretics, and comorbidity or history of hypertension, diabetes, CHD, stroke, COPD, and cancer.  Abbreviations: HR, hazard ratio; CI, confidential interval; BMI, body mass index; PIR, family income-to-poverty ratio; HEI, Healthy Eating Index; eGFR, estimated glomerular filtration rate; COPD, chronic obstructive pulmonary disease; CHD, coronary heart disease. |
| --- |
