# Supplementary material for: L-Shaped Association of Serum Chloride Level With All-Cause and Cause-Specific Mortality in American Adults: Population-Based Prospective Cohort Study
Source: JMIR Public Health Surveill. 2023 Nov 13;9:e49291. doi: 10.2196/49291 (PMC10682926; doi:10.2196/49291)
Supplement: Multimedia Appendix 3 [file publichealth_v9i1e49291_app3.doc]

| **Table S2. Subgroup analyses of the associations of serum chloride quartiles with CVD mortality for adults from the US National Health and Nutrition Examination Survey (NHANES) 1999-2018.** | | | | | | | |
| --- | --- | --- | --- | --- | --- | --- | --- |
| **Subgroup** | N | Q1(≤ 101.2) | Q2 (101.3, 103.2) | Q3 (103.3, 105.0) | Q4 (≥ 105.1) | *P* for trend | *P* for interaction |
|  |  | HR (95% CI) | HR (95% CI) | HR (95% CI) | HR (95% CI) |  |  |
| **Sex** |  |  |  |  |  |  | .81 |
| **Female** | 25632 | 1(ref) | 0.77(0.52,1.16) | 0.74(0.48,1.15) | 0.88(0.57,1.36) | .47 |  |
| **Male** | 25428 | 1(ref) | 0.56(0.42,0.75) | 0.49(0.35,0.68) | 0.59(0.40,0.87) | .006 |  |
| **Age** |  |  |  |  |  |  | .75 |
| **≤60** | 35508 | 1(ref) | 0.69(0.39,1.21) | 0.51(0.27,0.97) | 0.63(0.30,1.35) | .18 |  |
| **>60** | 15552 | 1(ref) | 0.58(0.46,0.73) | 0.60(0.46,0.79) | 0.74(0.54,0.99) | .03 |  |
| **Race** |  |  |  |  |  |  | .79 |
| **White** | 22199 | 1(ref) | 0.63(0.49,0.81) | 0.54(0.39,0.75) | 0.65(0.47,0.90) | .007 |  |
| **Non-white** | 28861 | 1(ref) | 0.69(0.42,1.14) | 0.67(0.43,1.05) | 0.83(0.51,1.34) | .38 |  |
| **Education** |  |  |  |  |  |  | .58 |
| **High school or below** | 23861 | 1(ref) | 0.60(0.46,0.79) | 0.47(0.33,0.66) | 0.55(0.38,0.79) | <.001 |  |
| **College or above** | 23630 | 1(ref) | 0.67(0.46,0.99) | 0.70(0.46,1.08) | 0.79(0.49,1.28) | .29 |  |
| **Marital status** |  |  |  |  |  |  | .92 |
| **Married** | 24924 | 1(ref) | 0.63(0.48,0.83) | 0.50(0.36,0.69) | 0.57(0.39,0.84) | .002 |  |
| **other** | 24116 | 1(ref) | 0.64(0.46,0.90) | 0.63(0.42,0.96) | 0.81(0.51,1.30) | .31 |  |
| **PIR** |  |  |  |  |  |  | .64 |
| **≤1.3** | 14912 | 1(ref) | 0.60(0.38,0.94) | 0.65(0.43,0.98) | 0.86(0.51,1.45) | .47 |  |
| **>1.3** | 31676 | 1(ref) | 0.66(0.51,0.85) | 0.55(0.40,0.75) | 0.62(0.43,0.88) | .005 |  |
| **BMI** |  |  |  |  |  |  | .29 |
| **≤28** | 26350 | 1(ref) | 0.53(0.41,0.69) | 0.57(0.41,0.80) | 0.59(0.41,0.87) | .003 |  |
| **>28** | 23827 | 1(ref) | 0.73(0.52,1.02) | 0.57(0.38,0.87) | 0.78(0.48,1.28) | .28 |  |
| **Smoker** |  |  |  |  |  |  | .63 |
| **Non-smoker** | 26271 | 1(ref) | 0.57(0.40,0.81) | 0.59(0.38,0.91) | 0.72(0.45,1.16) | .13 |  |
| **Smoker** | 22013 | 1(ref) | 0.66(0.50,0.88) | 0.54(0.38,0.76) | 0.62(0.43,0.91) | .01 |  |
| **Current drinker** |  |  |  |  |  |  | .995 |
| **No** | 14138 | 1(ref) | 0.68(0.47,0.98) | 0.58(0.37,0.91) | 0.67(0.41,1.10) | .10 |  |
| **Yes** | 29646 | 1(ref) | 0.64(0.48,0.84) | 0.58(0.42,0.79) | 0.69(0.48,1.00) | .03 |  |
| **HEI-2015** |  |  |  |  |  |  | .70 |
| **≤50** | 24430 | 1(ref) | 0.55(0.38,0.78) | 0.52(0.35,0.78) | 0.52(0.36,0.76) | <.001 |  |
| **>50** | 23604 | 1(ref) | 0.70(0.52,0.95) | 0.57(0.40,0.82) | 0.78(0.51,1.19) | .18 |  |
| **Physical activity** |  |  |  |  |  |  | .86 |
| **Inactive** | 13114 | 1(ref) | 0.69(0.55,0.86) | 0.68(0.46,1.00) | 0.79(0.52,1.21) | .18 |  |
| **Active** | 23689 | 1(ref) | 0.56(0.40,0.78) | 0.46(0.31,0.67) | 0.55(0.36,0.84) | .01 |  |
| **Diuretics usage** |  |  |  |  |  |  | .98 |
| **No** | 44379 | 1(ref) | 0.64(0.48,0.87) | 0.55(0.39,0.77) | 0.65(0.44,0.94) | .03 |  |
| **Yes** | 6632 | 1(ref) | 0.60(0.41,0.90) | 0.63(0.42,0.94) | 0.62(0.37,1.04) | .02 |  |
| **Hypertension** |  |  |  |  |  |  | .25 |
| **No** | 30501 | 1(ref) | 0.71(0.43,1.18) | 0.64(0.39,1.07) | 0.85(0.45,1.60) | .63 |  |
| **Yes** | 20545 | 1(ref) | 0.62(0.48,0.79) | 0.52(0.38,0.72) | 0.59(0.42,0.84) | .001 |  |
| **Diabetes** |  |  |  |  |  |  | .85 |
| **No** | 42533 | 1(ref) | 0.67(0.51,0.86) | 0.56(0.41,0.78) | 0.66(0.47,0.92) | .01 |  |
| **Yes** | 8523 | 1(ref) | 0.52(0.35,0.78) | 0.54(0.33,0.86) | 0.70(0.41,1.22) | .06 |  |
| **CHD** |  |  |  |  |  |  | .21 |
| **No** | 45305 | 1(ref) | 0.60(0.47,0.76) | 0.57(0.41,0.77) | 0.69(0.50,0.94) | .01 |  |
| **Yes** | 2040 | 1(ref) | 0.92(0.56,1.49) | 0.51(0.27,0.97) | 0.70(0.35,1.37) | .24 |  |
| **Stroke** |  |  |  |  |  |  | .99 |
| **No** | 45663 | 1(ref) | 0.63(0.50,0.80) | 0.55(0.42,0.73) | 0.65(0.48,0.89) | .005 |  |
| **Yes** | 1837 | 1(ref) | 0.56(0.21, 1.51) | 0.69(0.30, 1.62) | 0.94(0.37, 2.43) | .63 |  |
| **COPD** |  |  |  |  |  |  | .23 |
| **No** | 45581 | 1(ref) | 0.64(0.51,0.80) | 0.55(0.42,0.73) | 0.64(0.47,0.87) | .003 |  |
| **Yes** | 2009 | 1(ref) | 0.72(0.33, 1.59) | 0.61(0.26, 1.44) | 1.00(0.28, 3.54) | .91 |  |
| **Cancer** |  |  |  |  |  |  | .91 |
| **No** | 43107 | 1(ref) | 0.68(0.53,0.87) | 0.61(0.45,0.82) | 0.75(0.52,1.07) | .07 |  |
| **Yes** | 4404 | 1(ref) | 0.48(0.29,0.79) | 0.41(0.23,0.74) | 0.43(0.24,0.78) | .007 |  |
| **CKD** |  |  |  |  |  |  | .60 |
| **No** | 41298 | 1(ref) | 0.69(0.50,0.95) | 0.68(0.47,0.98) | 0.74(0.49,1.10) | .11 |  |
| **Yes** | 9215 | 1(ref) | 0.58(0.41,0.81) | 0.44(0.31,0.62) | 0.60(0.40,0.89) | .008 |  |

Data were calculated by svycoxph to fit a multivariate Cox proportional hazards model to data from a complex survey design, with adjustment of dichotomic sex, age, race, education, marital status, PIR, BMI, smoking, alcohol use, HEI-2015, physical activity, serum sodium, serum potassium, serum bicarbonate, usage of diuretics, and comorbidity or history of hypertension, diabetes, CHD, stroke, COPD, cancer and CKD except for the specific stratification variable. Test for trend was based on the variable containing the median value for each quartile. Interaction effect was evaluated by likelihood ratio test.

Abbreviations: HR: hazard ratio; CI, confidence interval; BMI, body mass index; PIR, family income-to-poverty ratio; HEI, Healthy Eating Index; COPD, chronic obstructive pulmonary disease; CHD, coronary heart disease; CKD, chronic kidney disease.
