# Supplementary material for: L-Shaped Association of Serum Chloride Level With All-Cause and Cause-Specific Mortality in American Adults: Population-Based Prospective Cohort Study
Source: JMIR Public Health Surveill. 2023 Nov 13;9:e49291. doi: 10.2196/49291 (PMC10682926; doi:10.2196/49291)
Supplement: Multimedia Appendix 6 [file publichealth_v9i1e49291_app6.doc]

| **Table S5. Survey-weighted multivariate analyses of the associations of categorical serum chloride with all-cause and cause-specific mortality after excluding participants who died in the first two years for adults from the US National Health and Nutrition Examination Survey (NHANES) 1999-2018.** | | | | | | | | | |  |
| --- | --- | --- | --- | --- | --- | --- | --- | --- | --- | --- |
|  | Q1(≤ 101.4) | | Q2 (101.5, 103.3) | | Q3 (103.4, 105.0) | | Q4 (≥ 105.1) | |  | |
|  | HR (95% CI) | | HR (95% CI) | *P*-value | HR (95% CI) | *P*-value | HR (95% CI) | *P*-value | *P* for trend | |
| **All-cause mortality** | | |  |  |  |  |  |  |  | |
| **Crude** | | 1(ref) | 0.60(0.55,0.66) | <.001 | 0.53(0.48,0.58) | <.001 | 0.60(0.54,0.67) | <.001 | <.001 | |
| **Model 1** | | 1(ref) | 0.73(0.67,0.79) | <.001 | 0.66(0.61,0.72) | <.001 | 0.74(0.67,0.81) | <.001 | <.001 | |
| **Model 2** | | 1(ref) | 0.76(0.67,0.86) | <.001 | 0.69(0.61,0.77) | <.001 | 0.74(0.65,0.84) | <.001 | <.001 | |
| **Model 3** | | 1(ref) | 0.81(0.70,0.93) | .003 | 0.74(0.64,0.86) | <.001 | 0.79(0.67,0.94) | .006 | .003 | |
| **CVD mortality** | | |  |  |  |  |  |  |  | |
| **Crude** | | 1(ref) | 0.58(0.50,0.67) | <.001 | 0.47(0.40,0.56) | <.001 | 0.58(0.48,0.70) | <.001 | <.001 | |
| **Model 1** | | 1(ref) | 0.68(0.60,0.78) | <.001 | 0.58(0.50,0.69) | <.001 | 0.71(0.59,0.84) | <.001 | <.001 | |
| **Model 2** | | 1(ref) | 0.62(0.51,0.75) | <.001 | 0.53(0.42,0.67) | <.001 | 0.69(0.55,0.87) | .002 | <.001 | |
| **Model 3** | | 1(ref) | 0.65(0.52,0.82) | <.001 | 0.56(0.42,0.73) | <.001 | 0.69(0.52,0.93) | .02 | .010 | |
| **Cancer mortality** | | |  |  |  |  |  |  |  | |
| **Crude** | | 1(ref) | 0.55(0.45,0.68) | <.001 | 0.60(0.50,0.73) | <.001 | 0.63(0.50,0.78) | <.001 | <.001 | |
| **Model 1** | | 1(ref) | 0.63(0.52,0.77) | <.001 | 0.69(0.56,0.83) | <.001 | 0.74(0.60,0.91) | .004 | .004 | |
| **Model 2** | | 1(ref) | 0.71(0.56,0.91) | .006 | 0.70(0.54,0.91) | .008 | 0.74(0.58,0.95) | .02 | .02 | |
| **Model 3** | | 1(ref) | 0.71(0.57,0.90) | .004 | 0.70(0.52,0.92) | .01 | 0.69(0.50,0.95) | .02 | .02 | |
| **Respiratory mortality** | | |  |  |  |  |  |  |  | |
| **Crude** | | 1(ref) | 0.45(0.35,0.58) | <.001 | 0.41(0.31,0.53) | <.001 | 0.39(0.29,0.53) | <.001 | <.001 | |
| **Model 1** | | 1(ref) | 0.54(0.42,0.69) | <.001 | 0.49(0.39,0.63) | <.001 | 0.48(0.36,0.64) | <.001 | <.001 | |
| **Model 2** | | 1(ref) | 0.64(0.39, 1.04) | .07 | 0.55(0.37, 0.80) | .002 | 0.51(0.32, 0.81) | .005 | .001 | |
| **Model 3** | | 1(ref) | 0.69(0.41, 1.15) | .16 | 0.56(0.37, 0.84) | .005 | 0.50(0.30, 0.84) | .009 | .004 | |

Data were calculated by svycoxph to fit a multivariate Cox proportional hazards model to data from a complex survey design. Test for trend was based on the variable containing the median value for each quartile.

| Model 1: Adjusted for sex, age, and race. |
| --- |
| Model 2: Adjusted for sex, age, race, education, marital status, PIR, BMI, smoking, alcohol use, HEI-2015, and physical activity. |
| Model 3: Adjusted for sex, age, race, education, marital status, PIR, BMI, smoking, alcohol use, HEI-2015, physical activity, serum sodium, serum potassium, serum bicarbonate, eGFR, usage of diuretics, and comorbidity or history of hypertension, diabetes, CHD, stroke, COPD, and cancer. |
| Abbreviations: HR, hazard ratio; CI, confidential interval; BMI, body mass index; PIR, family income-to-poverty ratio; HEI, Healthy Eating Index; eGFR, estimated glomerular filtration rate; COPD, chronic obstructive pulmonary disease; CHD, coronary heart disease. |
