# Supplementary material for: L-Shaped Association of Serum Chloride Level With All-Cause and Cause-Specific Mortality in American Adults: Population-Based Prospective Cohort Study
Source: JMIR Public Health Surveill. 2023 Nov 13;9:e49291. doi: 10.2196/49291 (PMC10682926; doi:10.2196/49291)
Supplement: Multimedia Appendix 7 [file publichealth_v9i1e49291_app7.doc]

| **Table S6. Survey-weighted multivariate analyses of the associations of categorical serum chloride with all-cause and cause-specific mortality after excluding participants with potential serum chloride outliers for adults from the US National Health and Nutrition Examination Survey (NHANES) 1999-2018.** | | | | | | | | | | | | |  |
| --- | --- | --- | --- | --- | --- | --- | --- | --- | --- | --- | --- | --- | --- |
|  | Q1(≤ 102.0) | Q2 (102.1, 103.6) | | | Q3 (103.7, 105.0) | | | | Q4 (≥ 105.1) | | |  |  |
|  | HR (95% CI) | HR (95% CI) | | *P*-value | HR (95% CI) | | *P*-value | | HR (95% CI) | | *P*-value | *P* for trend |  |
| **All-cause mortality** | |  | |  |  | |  | |  | |  |  |  |
| **Crude** | 1(ref) | 0.64(0.58,0.69) | | <.001 | 0.56(0.51,0.61) | | <.001 | | 0.62(0.56,0.69) | | <.001 | <.001 |  |
| **Model 1** | 1(ref) | 0.75(0.69,0.81) | | <.001 | 0.69(0.63,0.75) | | <.001 | | 0.75(0.69,0.82) | | <.001 | <.001 |  |
| **Model 2** | 1(ref) | 0.75(0.67,0.85) | | <.001 | 0.69(0.61,0.78) | | <.001 | | 0.73(0.64,0.83) | | <.001 | <.001 |  |
| **Model 3** | 1(ref) | 0.79(0.69,0.91) | | <.001 | 0.73(0.63,0.84) | | <.001 | | 0.77(0.65,0.91) | | .002 | <.001 |  |
| **CVD mortality** | |  | |  |  | |  | |  | |  |  |  |
| **Crude** | 1(ref) | 0.60(0.52,0.68) | | <.001 | 0.50(0.43,0.58) | | <.001 | | 0.58(0.49,0.68) | | <.001 | <.001 |  |
| **Model 1** | 1(ref) | 0.70(0.62,0.78) | | <.001 | 0.61(0.52,0.71) | | <.001 | | 0.70(0.60,0.82) | | <.001 | <.001 |  |
| **Model 2** | 1(ref) | 0.63(0.52,0.75) | | <.001 | 0.55(0.44,0.69) | | <.001 | | 0.67(0.54,0.83) | | .010 | <.001 |  |
| **Model 3** | 1(ref) | 0.65(0.52,0.80) | | <.001 | 0.56(0.43,0.74) | | <.001 | | 0.66(0.48,0.90) | | .08 | .005 |  |
| **Cancer mortality** | |  | |  |  | |  | |  | |  |  |  |
| **Crude** | 1(ref) | 0.60(0.49,0.72) | | <.001 | 0.63(0.53,0.76) | | <.001 | | 0.66(0.54,0.81) | | .012 | <.001 |  |
| **Model 1** | 1(ref) | 0.67(0.56,0.81) | | <.001 | 0.71(0.59,0.86) | | <.001 | | 0.77(0.63,0.94) | | .094 | .009 |  |
| **Model 2** | 1(ref) | 0.70(0.56,0.89) | | .003 | 0.69(0.54,0.88) | | .003 | | 0.72(0.57,0.91) | | .07 | .006 |  |
| **Model 3** | 1(ref) | 0.69(0.55,0.86) | | <.001 | 0.67(0.51,0.88) | | .004 | | 0.65(0.48,0.89) | | .14 | .006 |  |
| **Respiratory mortality** | |  | |  |  | |  | |  | |  |  |  |
| **Crude** | 1(ref) | 0.49(0.37,0.64) | | <.001 | 0.45(0.35,0.57) | | <.001 | | 0.41(0.31,0.55) | | <.001 | <.001 |  |
| **Model 1** | 1(ref) | 0.58(0.44,0.75) | | <.001 | 0.53(0.42,0.68) | | <.001 | | 0.50(0.38,0.66) | | <.001 | <.001 |  |
| **Model 2** | 1(ref) | 0.68(0.42, 1.10) | | 0.12 | 0.62(0.42, 0.90) | | .001 | | 0.55(0.34, 0.88) | | .01 | .007 |  |
| **Model 3** | 1(ref) | 0.69(0.42, 1.16) | | 0.16 | 0.58(0.38, 0.87) | | <.001 | | 0.47(0.28, 0.78) | | .004 | .002 |  |
| Data were calculated by svycoxph to fit a multivariate Cox proportional hazards model to data from a complex survey design. Test for trend was based on the variable containing the median value for each quartile. | | | | | | | | | | | | |  |
| Model 1: Adjusted for sex, age, and race. | | |  | | |  | |  | |  | |  |  |
| Model 2: Adjusted for sex, age, race, education, marital status, PIR, BMI, smoking, alcohol use, HEI-2015, and physical activity. | | | | | | | | | | | | |  |
| Model 3: Adjusted for sex, age, race, education, marital status, PIR, BMI, smoking, alcohol use, HEI-2015, physical activity, serum sodium, serum potassium, serum bicarbonate, eGFR, usage of diuretics, and comorbidity or history of hypertension, diabetes, CHD, stroke, COPD, and cancer. | | | | | | | | | | | | |  |
| Abbreviations: HR, hazard ratio; CI, confidential interval; BMI, body mass index; PIR, family income-to-poverty ratio; HEI, Healthy Eating Index; eGFR, estimated glomerular filtration rate; COPD, chronic obstructive pulmonary disease; CHD, coronary heart disease. | | | | | | | | | | | | |  |
