# Supplementary material for: L-Shaped Association of Serum Chloride Level With All-Cause and Cause-Specific Mortality in American Adults: Population-Based Prospective Cohort Study
Source: JMIR Public Health Surveill. 2023 Nov 13;9:e49291. doi: 10.2196/49291 (PMC10682926; doi:10.2196/49291)
Supplement: Multimedia Appendix 8 [file publichealth_v9i1e49291_app8.doc]

| **Table S7. Survey-weighted multivariate analyses of the associations of categorical serum chloride with all-cause and cause-specific mortality after excluding participants with possible hypoalbuminemia for adults from the US National Health and Nutrition Examination Survey (NHANES) 1999-2018.** | | | | | | | | | | | |  |
| --- | --- | --- | --- | --- | --- | --- | --- | --- | --- | --- | --- | --- |
|  | Q1(≤ 101.2) | | Q2 (101.3, 103.2) | | | Q3 (103.3, 105.0) | | Q4 (≥ 105.1) | |  | | |
|  | HR (95% CI) | | | HR (95% CI) | *P*-value | HR (95% CI) | *P*-value | HR (95% CI) | *P*-value | | *P* for trend | |
| **All-cause mortality** | | |  | |  |  |  |  |  | |  | |
| **Crude** | | 1(ref) | 0.59(0.54,0.64) | | <.001 | 0.51(0.47,0.56) | <.001 | 0.59(0.53,0.65) | <.001 | | <.001 | |
| **Model 1** | | 1(ref) | 0.71(0.66,0.77) | | <.001 | 0.65(0.60,0.70) | <.001 | 0.72(0.66,0.79) | <.001 | | <.001 | |
| **Model 2** | | 1(ref) | 0.74(0.65,0.83) | | <.001 | 0.67(0.59,0.75) | <.001 | 0.72(0.63,0.81) | <.001 | | <.001 | |
| **Model 3** | | 1(ref) | 0.78(0.68,0.89) | | <.001 | 0.71(0.62,0.81) | <.001 | 0.76(0.64,0.89) | <.001 | | <.001 | |
| **CVD mortality** | | |  | |  |  |  |  |  | |  | |
| **Crude** | | 1(ref) | 0.55(0.48,0.63) | | <.001 | 0.45(0.39,0.53) | <.001 | 0.55(0.46,0.65) | <.001 | | <.001 | |
| **Model 1** | | 1(ref) | 0.65(0.58,0.74) | | <.001 | 0.57(0.48,0.66) | <.001 | 0.67(0.57,0.80) | <.001 | | <.001 | |
| **Model 2** | | 1(ref) | 0.61(0.50,0.73) | | <.001 | 0.53(0.42,0.66) | <.001 | 0.66(0.53,0.82) | <.001 | | <.001 | |
| **Model 3** | | 1(ref) | 0.64(0.52,0.80) | | <.001 | 0.56(0.43,0.73) | <.001 | 0.68(0.51,0.91) | .009 | | .005 | |
| **Cancer mortality** | | |  | |  |  |  |  |  | |  | |
| **Crude** | | 1(ref) | 0.56(0.46,0.67) | | <.001 | 0.59(0.49,0.71) | <.001 | 0.63(0.51,0.77) | <.001 | | <.001 | |
| **Model 1** | | 1(ref) | 0.65(0.54,0.78) | | <.001 | 0.68(0.56,0.81) | <.001 | 0.75(0.62,0.91) | .003 | | .002 | |
| **Model 2** | | 1(ref) | 0.70(0.55,0.88) | | .002 | 0.67(0.52,0.85) | .001 | 0.71(0.57,0.90) | .005 | | .004 | |
| **Model 3** | | 1(ref) | 0.68(0.54,0.85) | | <.001 | 0.64(0.49,0.83) | <.001 | 0.63(0.47,0.84) | .002 | | .002 | |
| **Respiratory mortality** | | | | |  |  |  |  |  | |  | |
| **Crude** | | 1(ref) | 0.44(0.34,0.57) | | <.001 | 0.40(0.31,0.52) | <.001 | 0.38(0.28,0.50) | <.001 | | <.001 | |
| **Model 1** | | 1(ref) | 0.53(0.41,0.69) | | <.001 | 0.50(0.39,0.63) | <.001 | 0.47(0.36,0.62) | <.001 | | <.001 | |
| **Model 2** | | 1(ref) | 0.63(0.39, 1.02) | | .06 | 0.58(0.40, 0.84) | .004 | 0.52(0.32, 0.82) | .005 | | .002 | |
| **Model 3** | | 1(ref) | 0.69(0.41,1.15) | | .15 | 0.61(0.41,0.92) | .02 | 0.53(0.32,0.88) | .01 | | .007 | |

| Data were calculated by svycoxph to fit a multivariate Cox proportional hazards model to data from a complex survey design. Test for trend was based on the variable containing the median value for each quartile. |
| --- |
| Model 1: Adjusted for sex, age, and race. |
| Model 2: Adjusted for sex, age, race, education, marital status, PIR, BMI, smoking, alcohol use, HEI-2015, and physical activity. |
| Model 3: Adjusted for sex, age, race, education, marital status, PIR, BMI, smoking, alcohol use, HEI-2015, physical activity, serum sodium, serum potassium, serum bicarbonate, eGFR, usage of diuretics, and comorbidity or history of hypertension, diabetes, CHD, stroke, COPD, and cancer. |
|  |
| Abbreviations: HR, hazard ratio; CI, confidential interval; BMI, body mass index; PIR, family income-to-poverty ratio; HEI, Healthy Eating Index; eGFR, estimated glomerular filtration rate; COPD, chronic obstructive pulmonary disease; CHD, coronary heart disease. |
