# Supplementary material for: L-Shaped Association of Serum Chloride Level With All-Cause and Cause-Specific Mortality in American Adults: Population-Based Prospective Cohort Study
Source: JMIR Public Health Surveill. 2023 Nov 13;9:e49291. doi: 10.2196/49291 (PMC10682926; doi:10.2196/49291)
Supplement: Multimedia Appendix 9 [file publichealth_v9i1e49291_app9.doc]

| **Table S8. Survey-weighted multivariate analyses of the associations of categorical serum chloride with all-cause and cause-specific mortality after additionally adjusted of history of congestive heart failure and separately adjusted usage of potassium-sparing diuretics and potassium-wasting diuretics for adults from the US National Health and Nutrition Examination Survey (NHANES) 1999-2018.** | | | | | | | | |
| --- | --- | --- | --- | --- | --- | --- | --- | --- |
|  | Q1(≤ 101.2) | Q2 (101.3, 103.2) | | Q3 (103.3, 105.0) | | Q4 (≥ 105.1) | |  |
|  | HR (95% CI) | HR (95% CI) | P-value | HR (95% CI) | P-value | HR (95% CI) | P-value | P for trend |
| **All-cause mortality** | |  |  |  |  |  |  |  |
| **Model 3** | 1(ref) | 0.77(0.68,0.89) | <.001 | 0.72(0.63,0.82) | <.001 | 0.77(0.66,0.90) | .001 | <.001 |
| **Model 4** | 1(ref) | 0.78(0.68,0.89) | <.001 | 0.72(0.63,0.82) | <.001 | 0.77(0.66,0.91) | .002 | <.001 |
| **Model 5** | 1(ref) | 0.78(0.68,0.89) | <.001 | 0.72(0.62,0.82) | <.001 | 0.77(0.65,0.91) | .002 | <.001 |
| **CVD mortality** | |  |  |  |  |  |  |  |
| **Model 3** | 1(ref) | 0.63(0.51,0.79) | <.001 | 0.56(0.42,0.73) | <.001 | 0.67(0.50,0.89) | .006 | .004 |
| **Model 4** | 1(ref) | 0.64(0.52,0.80) | <.001 | 0.56(0.42,0.74) | <.001 | 0.67(0.50,0.90) | .007 | .004 |
| **Model 5** | 1(ref) | 0.64(0.52,0.80) | <.001 | 0.56(0.42,0.74) | <.001 | 0.67(0.50,0.89) | .007 | .004 |
| **Cancer mortality** | |  |  |  |  |  |  |  |
| **Model 3** | 1(ref) | 0.67(0.54,0.84) | <.001 | 0.65(0.49,0.85) | .002 | 0.65(0.48,0.87) | .004 | .004 |
| **Model 4** | 1(ref) | 0.67(0.54,0.84) | <.001 | 0.65(0.50,0.85) | .002 | 0.65(0.48,0.87) | .004 | .004 |
| **Model 5** | 1(ref) | 0.67(0.54,0.84) | <.001 | 0.65(0.49,0.85) | .002 | 0.65(0.48,0.87) | .004 | .003 |
| **Respiratory mortality** | |  |  |  |  |  |  |  |
| **Model 3** | 1(ref) | 0.68(0.41,1.13) | .14 | 0.59(0.40,0.88) | .009 | 0.51(0.31,0.84) | .008 | .004 |
| **Model 4** | 1(ref) | 0.69(0.41,1.16) | .16 | 0.61(0.41,0.91) | .02 | 0.50(0.30,0.83) | .008 | .004 |
| **Model 5** | 1(ref) | 0.69(0.41,1.15) | .16 | 0.61(0.41,0.90) | .01 | 0.50(0.30,0.83) | .007 | .003 |

| Data were calculated by svycoxph to fit a multivariate Cox proportional hazards model to data from a complex survey design. Test for trend was based on the variable containing the median value for each quartile.  Model 3: Adjusted for sex, age, race, education, marital status, PIR, BMI, smoking, alcohol use, HEI-2015, physical activity, serum sodium, serum potassium, serum bicarbonate, eGFR, usage of diuretics, and comorbidity or history of hypertension, diabetes, CHD, stroke, COPD, and cancer.  Model 4: Adjusted for sex, age, race, education, marital status, PIR, BMI, smoking, alcohol use, HEI-2015, physical activity, serum sodium, serum potassium, serum bicarbonate, eGFR, usage of diuretics, and comorbidity or history of hypertension, diabetes, CHD, stroke, COPD, cancer, and congestive heart failure.  Model 5: Adjusted for sex, age, race, education, marital status, PIR, BMI, smoking, alcohol use, HEI-2015, physical activity, serum sodium, serum potassium, serum bicarbonate, eGFR, usage of potassium-sparing diuretics, usage of non-potassium-sparing diuretics, and comorbidity or history of hypertension, diabetes, CHD, stroke, COPD, cancer, and congestive heart failure.  Abbreviation: HR, hazard ratio; CI, confidential interval; BMI, body mass index; PIR, family income-to-poverty ratio; HEI, Healthy Eating Index; eGFR, estimated glomerular filtration rate; COPD, chronic obstructive pulmonary disease; CHD, coronary heart disease. |
| --- |
