# Supplementary material for: L-Shaped Association of Serum Chloride Level With All-Cause and Cause-Specific Mortality in American Adults: Population-Based Prospective Cohort Study
Source: JMIR Public Health Surveill. 2023 Nov 13;9:e49291. doi: 10.2196/49291 (PMC10682926; doi:10.2196/49291)
Supplement: Multimedia Appendix 11 [file publichealth_v9i1e49291_app11.doc]

| **Table S9. Survey-weighted multivariate analyses of the associations of categorical serum chloride with all-cause and cause-specific mortality for adults after imputation of all missing covariates from the US National Health and Nutrition Examination Survey (NHANES) 1999-2018.** | | | | | | | | |
| --- | --- | --- | --- | --- | --- | --- | --- | --- |
|  | Q1(≤ 101.2) | Q2 (101.3, 103.2) | | Q3 (103.3, 105.0) | | Q4 (≥ 105.1) | |  |
|  | HR (95% CI) | HR (95% CI) | *P*-value | HR (95% CI) | *P*-value | HR (95% CI) | *P*-value | *P* for trend |
| **All-cause mortality** | |  |  |  |  |  |  |  |
| **Crude** | 1(ref) | 0.58(0.53,0.63) | <.001 | 0.51(0.47,0.56) | <.001 | 0.59(0.53,0.65) | <.001 | <.001 |
| **Model 1** | 1(ref) | 0.71(0.65,0.76) | <.001 | 0.65(0.60,0.70) | <.001 | 0.73(0.67,0.79) | <.001 | <.001 |
| **Model 2** | 1(ref) | 0.72(0.66,0.78) | <.001 | 0.66(0.61,0.71) | <.001 | 0.72(0.65,0.78) | <.001 | <.001 |
| **Model 3** | 1(ref) | 0.75(0.68,0.83) | <.001 | 0.70(0.63,0.76) | <.001 | 0.76(0.67,0.87) | <.001 | <.001 |
| **CVD mortality** | |  |  |  |  |  |  |  |
| **Crude** | 1(ref) | 0.54(0.47,0.62) | <.001 | 0.45(0.39,0.53) | <.001 | 0.55(0.46,0.65) | <.001 | <.001 |
| **Model 1** | 1(ref) | 0.65(0.58,0.74) | <.001 | 0.57(0.49,0.66) | <.001 | 0.68(0.58,0.80) | <.001 | <.001 |
| **Model 2** | 1(ref) | 0.65(0.57,0.74) | <.001 | 0.57(0.48,0.67) | <.001 | 0.67(0.57,0.78) | <.001 | <.001 |
| **Model 3** | 1(ref) | 0.67(0.57,0.79) | <.001 | 0.60(0.50,0.72) | <.001 | 0.68(0.55,0.85) | <.001 | <.001 |
| **Cancer mortality** | |  |  |  |  |  |  |  |
| **Crude** | 1(ref) | 0.55(0.46,0.66) | <.001 | 0.58(0.49,0.70) | <.001 | 0.63(0.51,0.76) | <.001 | <.001 |
| **Model 1** | 1(ref) | 0.64(0.53,0.76) | <.001 | 0.67(0.56,0.81) | <.001 | 0.75(0.62,0.90) | .003 | .002 |
| **Model 2** | 1(ref) | 0.68(0.57,0.82) | <.001 | 0.68(0.56,0.82) | <.001 | 0.76(0.63,0.93) | .006 | .004 |
| **Model 3** | 1(ref) | 0.68(0.57,0.81) | <.001 | 0.66(0.53,0.82) | <.001 | 0.69(0.53,0.89) | .005 | .004 |
| **Respiratory mortality** | |  |  |  |  |  |  |  |
| **Crude** | 1(ref) | 0.44(0.34,0.57) | <.001 | 0.40(0.31,0.51) | <.001 | 0.38(0.28,0.50) | <.001 | <.001 |
| **Model 1** | 1(ref) | 0.54(0.41,0.70) | <.001 | 0.49(0.39,0.62) | <.001 | 0.47(0.36,0.62) | <.001 | <.001 |
| **Model 2** | 1(ref) | 0.55(0.40,0.74) | <.001 | 0.47(0.37,0.60) | <.001 | 0.42(0.32,0.56) | <.001 | <.001 |
| **Model 3** | 1(ref) | 0.57(0.40,0.79) | <.001 | 0.47(0.35,0.62) | <.001 | 0.40(0.28,0.58) | <.001 | <.001 |

| Data were calculated by svycoxph to fit a multivariate Cox proportional hazards model to data from a complex survey design. Test for trend was based on the variable containing the median value for each quartile. |
| --- |
| Model 1: Adjusted for sex, age, and race. |
| Model 2: Adjusted for sex, age, race, education, marital status, PIR, BMI, smoking, alcohol use, HEI-2015, and physical activity. |
| Model 3: Adjusted for sex, age, race, education, marital status, PIR, BMI, smoking, alcohol use, HEI-2015, physical activity, serum sodium, serum potassium, serum bicarbonate, eGFR, usage of diuretics, and comorbidity or history of hypertension, diabetes, CHD, stroke, COPD, and cancer. |
| Abbreviations: HR, hazard ratio; CI, confidential interval; BMI, body mass index; PIR, family income-to-poverty ratio; HEI, Healthy Eating Index; eGFR, estimated glomerular filtration rate; COPD, chronic obstructive pulmonary disease; CHD, coronary heart disease. |
