# Supplementary material for: L-Shaped Association of Serum Chloride Level With All-Cause and Cause-Specific Mortality in American Adults: Population-Based Prospective Cohort Study
Source: JMIR Public Health Surveill. 2023 Nov 13;9:e49291. doi: 10.2196/49291 (PMC10682926; doi:10.2196/49291)
Supplement: Multimedia Appendix 12 [file publichealth_v9i1e49291_app12.doc]

| **Table S10. E-value analysis for categorical serum chloride with all-cause and cause-specific mortality in adult participants from NHANES 1999-2018.** | | |
| --- | --- | --- |
|  | HR (95%CI) | E-value |
| **All-cause mortality** |  |  |
| **Q1(≤ 101.2)** | 1.00(ref) | ref |
| **Q2 (101.3, 103.2)** | 0.77(0.67,0.89) | 1.92 |
| **Q3 (103.3, 105.0)** | 0.72(0.63,0.82) | 2.12 |
| **Q4 (≥ 105.1)** | 0.77(0.65,0.90) | 1.92 |
| **CVD mortality** |  |  |
| **Q1(≤ 101.2)** | 1.00(ref) | ref |
| **Q2 (101.3, 103.2)** | 0.63(0.51,0.79) | 2.55 |
| **Q3 (103.3, 105.0)** | 0.56(0.43,0.73) | 2.97 |
| **Q4 (≥ 105.1)** | 0.67(0.50,0.89) | 2.34 |
| **Cancer mortality** |  |  |
| **Q1(≤ 101.2)** | 1.00(ref) | ref |
| **Q2 (101.3, 103.2)** | 0.67(0.54,0.84) | 2.35 |
| **Q3 (103.3, 105.0)** | 0.65(0.50,0.85) | 2.45 |
| **Q4 (≥ 105.1)** | 0.65(0.48,0.87) | 2.45 |
| **Respiratory mortality** |  |  |
| **Q1(≤ 101.2)** | 1.00(ref) | ref |
| **Q2 (101.3, 103.2)** | 0.68(0.41,1.13) | NA |
| **Q3 (103.3, 105.0)** | 0.59(0.40,0.88) | 2.78 |
| **Q4 (≥ 105.1)** | 0.51(0.31,0.84) | 3.33 |

HR and 95% CIs were calculated by svycoxph to fit a multivariate Cox proportional hazards model to data from a complex survey design, with adjustment of sex, age, race, education, marital status, PIR, BMI, smoking, alcohol use, HEI-2015, physical activity, serum sodium, serum potassium, serum bicarbonate, eGFR, usage of diuretics, and comorbidity or history of hypertension, diabetes, CHD, stroke, COPD, and cancer.

Abbreviations: HR, hazard ratio; CVD, cardiovascular disease
